# Bioresorbable, wireless, passive sensors for continuous pH measurements and early detection of gastric leakage

**DOI:** 10.1126/sciadv.adj0268

**Published:** 2024-04-19

**Authors:** Shuo Li, Di Lu, Shupeng Li, Jiaqi Liu, Yameng Xu, Ying Yan, Jorge Zárate Rodriguez, Hedan Bai, Raudel Avila, Shuming Kang, Xinchen Ni, Haiwen Luan, Hexia Guo, Wubin Bai, Changsheng Wu, Xuhao Zhou, Ziying Hu, Mitchell A. Pet, Chet W. Hammill, Matthew R. MacEwan, Wilson Z. Ray, Yonggang Huang, John A. Rogers

**Affiliations:** ^1^ Querrey Simpson Institute for Bioelectronics, Northwestern University, Evanston, IL 60208, USA.; ^2^ School of Microelectronics, University of Science and Technology of China, Hefei, Anhui 230026, China.; ^3^ Department of Mechanical Engineering, Northwestern University, Evanston, IL 60208, USA.; ^4^ The Institute of Materials Science and Engineering, Washington University in St. Louis, St. Louis, MO 63130, USA.; ^5^ Department of Neurosurgery, Washington University School of Medicine, St. Louis, MO 63110, USA.; ^6^ Department of Surgery, Washington University School of Medicine, St. Louis, MO 63110, USA.; ^7^ Department of Materials Science and Engineering, Northwestern University, Evanston, IL 60208, USA.; ^8^ Department of Applied Physical Sciences, University of North Carolina at Chapel Hill, Chapel Hill, NC 27599, USA.; ^9^ Department of Materials Science and Engineering, National University of Singapore, Singapore 117575, Singapore.; ^10^ Department of Chemistry, Northwestern University, Evanston, IL 60208, USA.; ^11^ Department of Civil and Environmental Engineering, Northwestern University, Evanston, IL 60208, USA.; ^12^ Department of Biomedical Engineering, Northwestern University, Evanston, IL 60208, USA.; ^13^ Department of Neurological Surgery, Feinberg School of Medicine, Northwestern University, Chicago, IL 60611, USA.

## Abstract

Continuous monitoring of biomarkers at locations adjacent to targeted internal organs can provide actionable information about postoperative status beyond conventional diagnostic methods. As an example, changes in pH in the intra-abdominal space after gastric surgeries can serve as direct indicators of potentially life-threatening leakage events, in contrast to symptomatic reactions that may delay treatment. Here, we report a bioresorbable, wireless, passive sensor that addresses this clinical need, designed to locally monitor pH for early detection of gastric leakage. A pH-responsive hydrogel serves as a transducer that couples to a mechanically optimized inductor-capacitor circuit for wireless readout. This platform enables real-time monitoring of pH with fast response time (within 1 hour) over a clinically relevant period (up to 7 days) and timely detection of simulated gastric leaks in animal models. These concepts have broad potential applications for temporary sensing of relevant biomarkers during critical risk periods following diverse types of surgeries.

## Teaser

Fully bioresorbable, passive sensor enables continuous, rapid monitoring of pH for early detection of gastric leakage.

## INTRODUCTION

Anastomotic leaks represent significant complications associated with gastric surgeries, with reported rates as high as 1.3 to 21% in surgery for gastric tumor excision and 0 to 5.6% in weight-loss surgery (over 250,000 surgeries performed annually in the United States), depending on specific types of surgeries that produce different gastric wall ischemia (*1*, *2*). Consequences of leaks include sepsis, multi-organ system failure, and other adverse reactions (*3*), with associated increases in rates of complications and of mortality by factors of three and four, respectively (*4*). Although prompt, accurate diagnosis is crucial to the effective care of these patients, leaks often remain undetected until after the development of outward symptomatic signs such as severe inflammation, abdominal pain, or persistent tachycardia (*5*). Such clinical manifestations are often delayed by days after the start of the leak and they can be subtle for certain types of patients, in particular the obese, as evidence indicates that about half of those who suffer anastomotic leakage are often asymptomatic (*3**,* *6*). The conventional method for screening anastomotic leak relies on radiological investigations of the abdomen [e.g., upper gastrointestinal (GI) series and computerized tomography scans] when the quality or output of the gastric drainage changes (*7*). The unmet clinical need is, therefore, in a minimally invasive system capable of detecting gastric leaks early in the postoperative course before the development of clinical sequelae. As the leaked contents from the stomach feature high acidity compared to other abdominal fluids due to the high hydrogen ion concentration in gastric fluid, an implantable, wireless pH sensor may act as the most effective approach for timely detection.

Traditional means for pH measurements are poorly matched for such purposes, as they involve components that are neither biocompatible nor well suited for temporary implants. For example, chromatic acid-base indicator dyes are often toxic and hazardous. Electrochemical potentiometers require rigid glass/reference electrodes and bulky onboard electronics. Both types of approaches demand materials and/or hardware that must be surgically removed after a period of need set by the timescale for significant risk of leaks, typically a few days to a week after the surgery (*8*). Recently reported classes of bioresorbable electronic sensor systems have great potential in this context (*9*–*13*). Such devices may be implanted during the surgery, serving as a temporary but enabling technology to realize wireless, continuous pH sensing at target locations inside the body. Natural processes of disintegration, dissolution, and bioresorption then eliminate the devices after they are no longer needed, eventually self-clearing into benign end products via normal metabolic mechanisms, without the need for an extraction surgery (*14*–*16*).

Although bioresorbable electronic sensors of biophysical parameters such as pressure, temperature, and flow rate are well established (*10*, *12*, *16*–*22*), those with capabilities to respond to biochemical changes are relatively underexplored (*10*, *23*, *24*). Materials that can transduce biochemical markers into physical changes are, therefore, important to consider. Recent advances in responsive hydrogels offer potential in point-of-care detection and medical diagnostics (*25*–*28*), owning to their excellent mechanical compliance, biocompatibility, biodegradability, and selective responsiveness to physical (temperature, light, and pressure) (*29*–*31*), chemical (pH, glucose, and ionic strengths) (*32*, *33*), and/or biological (antigens/antibodies and enzymes) stimuli (*34*, *35*). Alterations in the conformations of the polymers that constitute these hydrogels effectively transduce such stimuli into changes in dimensions, optical characteristics, or electrical properties, each of which can be quantitatively captured by various physical sensors (*26*). For implantable biosensing purposes such as real-time, potential clinical monitoring of gastric leaks, however, combining pH-responsive, biodegradable hydrogels for sensing with wireless, bioresorbable electronic systems for data communication would serve as a promising engineering approach.

Here, we report a collection of materials and device architectures that support wireless sensing of pH with fast response times, in a fully bioresorbable system that exploits passive analog wireless communication schemes. Functionalizing the poly(ethylene glycol) polymer backbone with pH-sensitive tertiary amine groups yields a highly selective pH-responsive hydrogel that is both biodegradable and mechanically robust. This material acts as a supporting matrix for an inductor-capacitor (LC) resonant circuit that efficiently converts dimensional changes into shifts in the resonant frequency, with magnitudes that can be quantified accurately by inductive coupling to an external reader device. Mechanics simulations guide choices in optimal geometries of the hydrogel and the circuit components, specifically the inductor. Systematic experiments in both animal models and in vitro setups validate all attributes of the device for continuous monitoring of changes in pH. Histological investigations along with assays of blood chemistry and complete blood count establish the biocompatibility and bioresorbability of this technology.

## RESULTS

### Device design, operating principle, and envisioned medical use

**Fig. 1.**
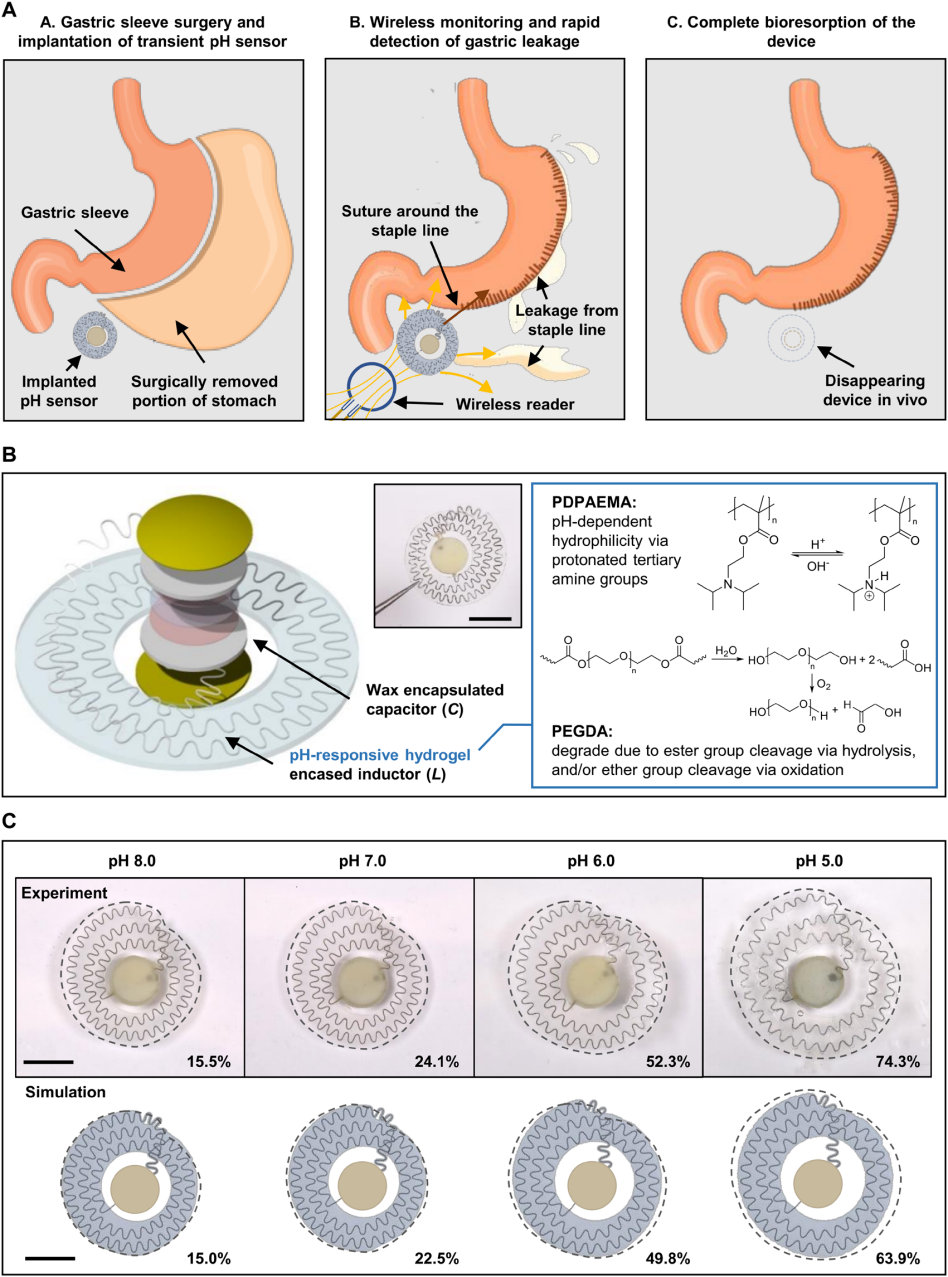
A bioresorbable, flexible, and passive wireless pH sensor. (**A**) Schematic illustrations of the envisioned medical use of the implanted device, including postoperative wireless monitoring of gastric leakage after LSG surgery followed by complete bioresorption after a postsurgical recovery period. (**B**) Materials and design features of the LC circuit that forms the basis of the sensor. Left: Exploded view schematic illustration showing the active sensing component, a serpentine spiral inductor encased in a pH-responsive hydrogel, and a wax encapsulated capacitor. Inset: Photograph of an assembled device. Right: Chemical structures of the polymers that comprise the hydrogel, along with the chemistry of its pH response and biodegradation. Scale bar, 10 mm. (**C**) pH responses of the device. Optical images (top row) and simulation results (bottom row) of the physical expansion of the sensors following immersion in aqueous buffered solutions with different pH after 2 hours. Magnitudes of the area expansion of hydrogel/inductor structures are labeled to the bottom right corner of each image. Scale bars, 10 mm.

Figure 1A depicts an envisioned medical use of this bioresorbable LC circuit as an implantable, wireless sensor of pH. The device in this case provides the ability to monitor for potential leak of gastric fluid during the critical risk period following a laparoscopic sleeve gastrectomy (LSG). This bariatric procedure removes 75 to 80% of the stomach, shaping the remaining part into a tube or “gastric sleeve” (*36*). As a commonly reported postoperative complication, gastric fistulas or staple line dehiscence may develop, inducing an outflow of GI content around the stomach, typically at some point during an approximately 7-day recovery period following the LSG (*37*). Placing a wireless readout coil over the abdomen allows wireless detection of the resonant frequency (*f*_s_) of the sensor circuit, which depends on the acidity of the surrounding biofluid. Before externally observable clinical signatures or symptoms, any leaked gastric fluids (pH ~1 to 3) will permeate into the hydrogel of the sensor, leading to swelling and associated rapid (<1 hour) shift in *f*_s_ as an alarm to prompt surgical intervention to make an additional anastomosis. The materials selection and design features are such that the device disintegrates and naturally dissolves, in a biocompatible fashion without residue, after the recovery period has elapsed, thereby eliminating the risks and costs associated with an additional surgery for extraction.

The schematic illustration in Fig. 1B (left, inset) presents the design of this wireless bioresorbable pH sensor. The sensitive component is a pH-responsive hydrogel that encases an inductor (inductance *L*) in the form of a filamentary serpentine spiral structure. The hydrogel is a poly[2-(diisopropylamino)ethyl methacrylate] and poly(ethylene glycol)diacrylate (PDPAEMA-PEGDA) copolymer synthesized via a one-step free radical photopolymerization. As shown in Fig. 1B (right), PDPAEMA serves as the pH-responsive moiety with a p*K*_a_ (where *K*_a_ is the acid dissociation constant) around 6.3 (*38*). At pH values lower than the p*K*_a_, the protonated tertiary amine units lead to electrostatic repulsive forces and osmotic pressure within the polymer network, thereby inducing rapid swelling of the hydrogel (*39*, *40*). The pH-responsive PDPAEMA-PEGDA degrades in biofluids through hydrolysis of the constituting ester groups (*41*). The degradation of PEGDA can also occur through ether cleavage of oxidation or through a combination of both hydrolysis and oxidation (*42**,* *43*). The degraded derivatives are nontoxic, water soluble, and excretable from the body.

The inductive coil consists of a thin, narrow, serpentine structure of Zn coated with a thin layer of bioresorbable wax. The geometry minimizes the effective Young’s modulus of the structure, to enable relatively unconstrained area expansion upon swelling of the surrounding hydrogel matrix (*44*). The parallel-plate capacitor (capacitance *C*) uses a film of bioresorbable poly(lactide-co-glycolide) (PLGA) as the dielectric layer sandwiched between a pair of Zn electrodes and thermally bonded via a poly(vinyl alcohol) (PVA) adhesive. This stack electrically connects to the inductive coil through small deposits of a bioresorbable conductive paste that consists of a mixture of candelilla wax and W microparticles [for details, see (*45*)]. A blend of beeswax and candelilla wax serves as a water barrier to prevent drifts in the response caused by permeation of biofluids (*18*). The overall device is compact (diameter, ~25 mm; thickness, <700 μm) and lightweight (~0.27 g). Details associated with the fabrication procedures and dimensions appear in Materials and Methods and figs. S1 and S2.

**Fig. 2.**
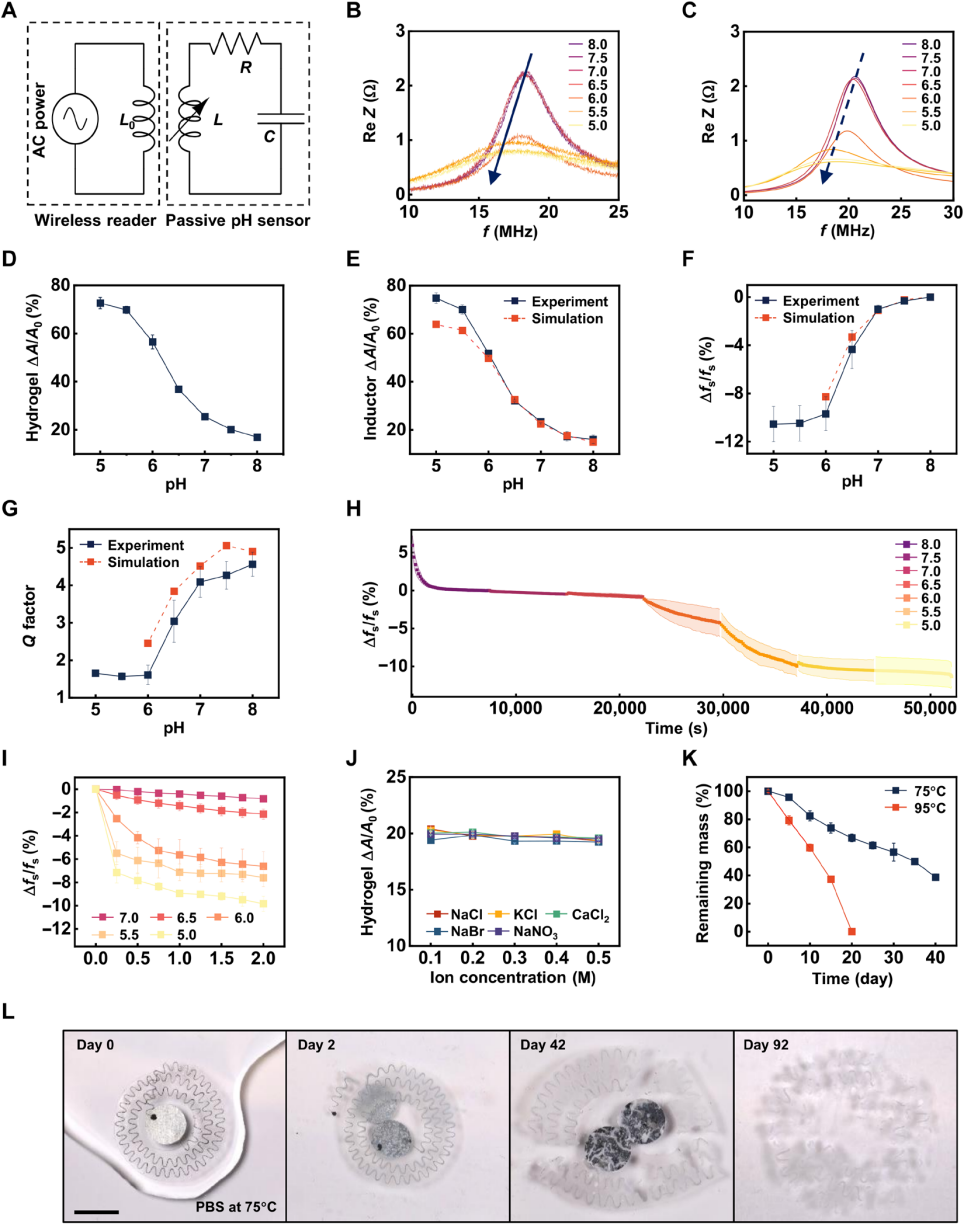
pH sensing mechanisms and properties of the materials and devices. (**A**) Equivalent circuit for the passive LC-resonance sensor and the wireless readout system. (**B**) Wireless measurements of the real part of the impedance (Re *Z*) as a function of frequency (*f*) after consecutive immersion of the sensor in pH buffers (2 hours in each). (**C**) Simulation results of the Re *Z* as a function of *f*. (**D**) Measurements of the area expansion (Δ*A*/*A*_0_) of the pH-responsive hydrogel as a function of pH after 2 hours of immersion. (**E**) Experimental measurements and simulation results for the Δ*A*/*A*_0_ as a function of pH. (**F**) Experimental measurements and simulation results of the pH dependence of the variation in resonant frequency (Δ*f*_s_/*f*_s_). (**G**) Experimental measurements and simulation results of the pH dependence of the *Q* factor. (**H**) Continuous measurements of Δ*f*_s_/*f*_s_ as a function of time, starting from the dry state, followed by the immersion in citrate buffer solutions of descending pH. (**I**) Measurements of Δ*f*_s_/*f*_s_ as a function of time, starting from the PBS-swollen state (pH = 7.4). (**J**) Measurements of hydrogel Δ*A*/*A*_0_ as a function of ion concentration for selected common ions. (**K**) Measured changes in weight for the pH-responsive hydrogels as a function of time of immersion in PBS, at temperatures of 75° and 95°C. (**L**) Photographs of the accelerated dissolution of a pH sensor in PBS (PBS solution refreshed every 5 days). The biodegradable wax encapsulation is not included in this example, to improve visualization of the dissolution of the capacitor. Scale bar, 10 mm.

Figure 1C presents photographs (top row) and simulation results (bottom row) associated with the area expansion of the pH-responsive hydrogel with an embedded inductor following immersion in citrate buffer solutions [citric acid (CA)–trisodium citrate, 0.1 M] of different pH. The images show the pH dependence of the hydrogel expansion and associated deformation of the inductor. The dotted lines define the perimeters of the hydrogels in the top row, mapped to that of the simulation sketches, demonstrating excellent agreement (deviation = −4.97% ± 1.47%) for nearly all cases (except for pH 5.0, refer to Fig. 2E for detailed results).

Figure 2A summarizes the operating principle of the devices. As highlighted in Fig. 1C, a drop in the pH of the surrounding fluid to values below the p*K*_a_ of the hydrogel triggers swelling that physically expands the inductor and increases its inductance. Connecting this variable inductor to a fixed capacitor in series forms an LC circuit that resonates at a frequency $f_{s}\left(\text{pH}\right)=\frac{1}{2\pi \sqrt{L\left(\text{pH}\right)C}}$ . Characterizations of both *L* and *C* separately further validate this statement (fig. S3). Near-field magnetic coupling to a readout coil with inductance *L*_0_ enables real-time, remote sensing of *f*_s_ when connected to a vector network analyzer (VNA). As shown in Eq. 1, the peak of the real part of the impedance (Re *Z*) as a function of frequency *f* across *L*_0_ defines the value of *f*_s_ (*46*).

$$\text{Re}\;Z=\frac{kf^{2}}{1+Q^{2}{\left(f/f_{s}-f_{s}/f\right)}^{2}}$$ (1)

where *Q* ( $Q=\frac{1}{R}\sqrt{\frac{L}{C}}$ ) is the quality factor (or *Q* factor) and *k* is the coupling constant, which depends on the distance and relative angle between *L* and *L*_0_. As illustrated in Fig. 2B, after each 2-hour consecutive session of immersion in citric buffer solutions, the resonant frequency *f*_s_ of the sensor approximately equals to the frequency corresponding to the maximum of Re *Z*, monotonically decreases with decreasing pH, as expected based on the expanded geometry of the spiral coil. The plot also indicates a rapid reduction in the amplitude of Re *Z*, consistent with a substantial reduction in the *Q* factor (for more detailed explanation, see the discussion of Fig. 2H). Numerical simulations of Re *Z* of the sensor as a function of pH show similar trends (Fig. 2C).

### Design and characterization of materials

Detailed characterization of the swelling/deswelling behavior for the PDPAEMA-PEGDA copolymer hydrogel reveals quantitative relationships between pH, inductor/hydrogel deformation, and resonance parameters (*f*_s_ and *Q*). Figure 2D shows the area expansion of the hydrogel (thickness = 400 μm) from dry to swollen states in citrate buffer solutions (from pH 8.0 to pH 5.0, to cover the range of pH values relevant for this application) after 2 hours of immersion (the optimization of hydrogel thickness is discussed in the caption of fig. S4). Finite element analysis (FEA) captures the deformation of the hydrogel with an embedded serpentine inductor, where the hydrogel area expansion data (shown in Fig. 2D) provide essential input (refer to Materials and Methods for details). Without any parameter fitting, Fig. 2E shows that the simulated area expansion of the inductor/hydrogel structure agrees well with that from experiments in the range of pH between 8.0 and 6.0 (deviation = −2.25% ± 3.87%). For pH values below 6.0, the hydrogel undergoes deformations beyond its elastic limit, leading to the formation of cracks that are difficult to simulate (fig. S5). Electromagnetic FEA determines the relative change in resonant frequency (Δ*f*_s_/*f*_s_) due to the area expansion of the inductor/hydrogel. Again, without any parameter fitting, the simulation results match well (deviation = −14.91% ± 17.49%) with those from experiments (Fig. 2F). The results shown in Fig. 2G quantitatively capture the decrease in the *Q* factor, consistent with Fig. 2 (B and C). This decline follows from the formation of –NH(R)^2+^, R = CH(CH3)_2_, and mobile anions in the DPAEMA (Fig. 1B, right) that causes increased electromagnetic dissipation as the pH decreases. Simple calculations based on Fickian diffusion of hydrogen ions (representative diffusivity of hydrogen ions through the hydrogel: 1 × 10^−6^ to 1 × 10^−5^ mm^2^ s^−1^) suggest an equilibrium time of 4000 to 40,000 s (for detailed modeling, refer to note S1) (*47*). Further examination of the time dependence of ∆*f*_s_/*f*_s_ starting from the dry state (Fig. 2H and fig. S6) verifies that the response time for pH sensing is at the similar level as that of hydrogel swelling, as expected (*48*). Intermittent wireless reading of ∆*f*_s_/*f*_s_ from the phosphate-buffered saline (PBS) swollen state characterizes the time dependence of the response of the sensor to pH (Fig. 2I and fig. S7) and thus serves as the basis for pH measurements in the following context.

Ion selectivity is a critical aspect of any pH sensor. Conventional potentiometric pH meters include a glass electrode that is highly selective to hydrogen ions (*49*). In general, pH-responsive hydrogels also have high ion selectivity (*50*). As demonstrated in Fig. 2J, the hydrogel used in this work exhibits minimal response to common ions (Na^+^, K^+^, Ca^2+^, Cl^−^, Br^−^, and NO_3_^−^) across a broad range of physiologically relevant concentrations in simulated biofluids. In addition, the pH-dependent swelling does not rely on buffer type (citrate or phosphate), as shown in fig. S8. The selectivity likely results from the specific binding of the amine group in DPAEMA to H^+^, a well-studied mechanism in tertiary amine-based methacrylate polymers (*51*). Other tests demonstrate robust operation in the context of other potentially confounding effects expected in practical applications. Specifically, both experimental measurements and simulation results indicate that the response of the device does not change with deformations caused by natural movements (e.g., applied force, in-plane uniaxial stretching, out-of-plane bending, and twisting) and that it is minimally affected by temperature (figs. S9 to S11), over relevant ranges expected for implants.

Accelerated evaluations of the processes of bioresorption involve immersion of the constituent materials of the device in PBS solution at elevated temperatures. At 95°C, the hydrogel material completely dissolves within 20 days, whereas the dissolution rate drops to ~1/4 at 75°C (Fig. 2K). On the basis of an Arrhenius scaling relationship ( $k=Ae^{-\frac{E_{a}}{k_{B}T}}$ ), where *A* is the pre-exponential factor, *E*_a_ is the activation energy, assumed to be independent of temperature in the range of 37° to 90°C, and *k*_B_ is the Boltzmann constant, the projected time of dissolution for the hydrogel at 37°C is ~700 days (fig. S12). Previous studies confirm the biodegradation and bioresorption of all other materials (metal, polymers, and natural wax). For examples, zinc reacts with water [Zn + 2H_2_O → Zn(OH)_2_ + H_2_] to yield soluble hydroxides (*14*). Tungsten in the conductive wax paste oxidizes in water to produce a soluble acid (2W + 2H_2_O + O_2_ → 2H_2_WO_4_) (*14*). PLGA degrades into lactic acid and glycolic acid; both species enter the tricarboxylic acid cycle in vivo and are metabolized and eventually eliminated from the body as carbon dioxide and water (*52*). PVA is nontoxic, biologically inert, and soluble in water (*53*). Wax is biocompatible and gradually degrades in vivo (*45*). Figure 2L shows accelerated dissolution of the entire device (75°C), where no trace materials remain in PBS at day 92.

### Optimization of sensor design via mechanics simulation

A key requirement of the inductor design is that it should not substantially inhibit the free expansion of the hydrogel. Specifically, the geometry of the inductor minimizes the mechanical compliance, while balancing the requirement of sufficient thickness to ensure low resistance and corresponding high *Q* factor. The filamentary serpentine layout converts strains induced through hydrogel expansion into microscale buckling and twisting motions along the lengths of the Zn traces (*44*), to yield an effective modulus given by Eq. 2 (refer to note S2 for more details) (*54*)

$$E_{\text{eff}}=\frac{16\;{\mathrm{Eb}}^{3}}{\lambda^{2}H\left[4{\left(\frac{2H}{\lambda }-1\right)}^{3}+6\pi {\left(\frac{2H}{\lambda }-1\right)}^{2}+24\left(\frac{2H}{\lambda }-1\right)+3\pi \right]}$$ (2)

where *E* is the Young’s modulus of the material in bulk (i.e., Zn), *b* is the width, λ is the periodic length of each serpentine unit cell, and *H* is the height of the serpentine (parameter definitions see fig. S13). Considering the need to maintain compact size and to support high *Q* factor, the effective modulus *E*_eff_ can reach as low as 3.9 MPa for a thickness *h*_0_ = 0.1 mm, with width *b*_0_ = 0.1 mm, serpentine height *H*_0_ = 2 mm, and unit cell length λ_0_ = 2.1 mm. This value of *E*_eff_ is well below that of the Zn (*E*_Zn_ = 108 GPa) and the hydrogel (*E*_hydrogel_ = 6.5 to 9.8 MPa in different pH buffers; fig. S14); the latter allows for relatively unconstrained expansion of the hydrogel.

**Fig. 3.**
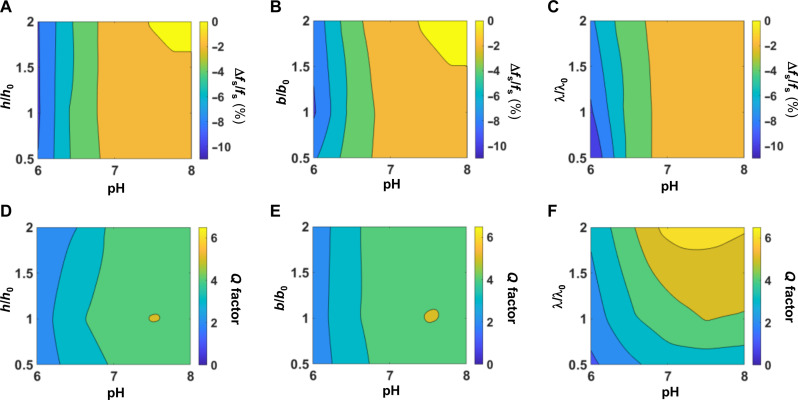
Zn inductive coil design guided by simulations of the mechanical properties. (**A**) Surface contour plots of the simulated Δ*f*_s_/*f*_s_ as a function of the thickness of the Zn serpentine inductor (*h*/*h*_0_, where *h*_0_ = 0.1 mm) and pH. Successive contour lines represent an interval of 2%. (**B**) Simulation results for the width of the inductor (*b*/*b*_0_, where *b*_0_ = 0.1 mm), analogous to that in (A). (**C**) Simulation results for the unit cell length of the inductor (λ/λ_0_, where λ_0_ = 1.05 mm), analogous to those in (A) and (B). (**D**) Surface contour plots of the simulated *Q* factor as a function of *h*/*h*_0_ and pH. Successive contour lines represent an interval of 2. (**E**) Simulation results for width of the inductor (*b*/*b*_0_, where *b*_0_ = 0.1 mm), analogous to that in (D). (**F**) Simulation results for the unit cell length of the inductor (λ/λ_0_, where λ_0_ = 1.05 mm), analogous to those in (D) and (E). *h*_0_, *b*_0_, and λ_0_ are the final parameters used for the devices used here.

These parameters (*h*_0_, *b*_0_, and λ_0_) also yield excellent electromagnetic performance, specifically, sufficiently large Δ*f*_s_/*f*_s_ (compared to those due to parasitic effects associated with deformations, drifts, misalignments, etc.) and *Q* factor (to suppress measurement error), to support good sensitivity and consistent wireless reading of leakage. Simulation results for both parameters as a function of thickness *h*, width *b*, and unit cell length λ are illustrated in two-dimensional (2D) contour plots (Fig. 3 A and D, B and E, and C and F, respectively; the dependence on the serpentine height *H* will be discussed later). Figure 3 (A and B) suggests that Δ*f*_s_/*f*_s_ is approximately independent of thickness and width; the combination of *h*_0_ and *b*_0_ is ideal as it leads to sufficiently low elastic modulus based on the mechanics analysis as discussed above. Figure 3 (C and F) demonstrates an opposite dependence of Δ*f*_s_/*f*_s_ and *Q* factor on unit cell length: There is a raise in Δ*f*_s_/*f*_s_ and a reduction in *Q* factor as λ/λ_0_ decreases, so λ_0_ represents the best compromise. Also, the magnitude of Δ*f*_s_/*f*_s_ is positively correlated with the change in inductance, which in turn is positively associated with the area expansion of the inductor, and thus implies a negative correlation with the effective modulus *E*_eff_. According to our governing equation (Eq. 2), *E*_eff_ is independent of *h* and positively associated with λ, suggesting again that Δ*f*_s_/*f*_s_ does not depend strongly on *h* (Fig. 3A) and that Δ*f*_s_/*f*_s_ correlates negatively with λ (Fig. 3C). Further decreasing λ, however, increases the length of the coil, which increases its electrical resistance and decreases *Q* factor. The effective modulus *E*_eff_ also increases with *b*, but the inductance itself decreases with the width (*55*). These opposing effects balance one another, leading to a weak dependence of Δ*f*_s_/*f*_s_ on *b* as shown in Fig. 3B. Last, a compliant serpentine structure favors a large height *H*. As a result, *H*_0_ adopts the largest value that avoids overlap between neighboring inner and outer loops.

### Evaluation of sensing performance in vitro and in animal models

**Fig. 4.**
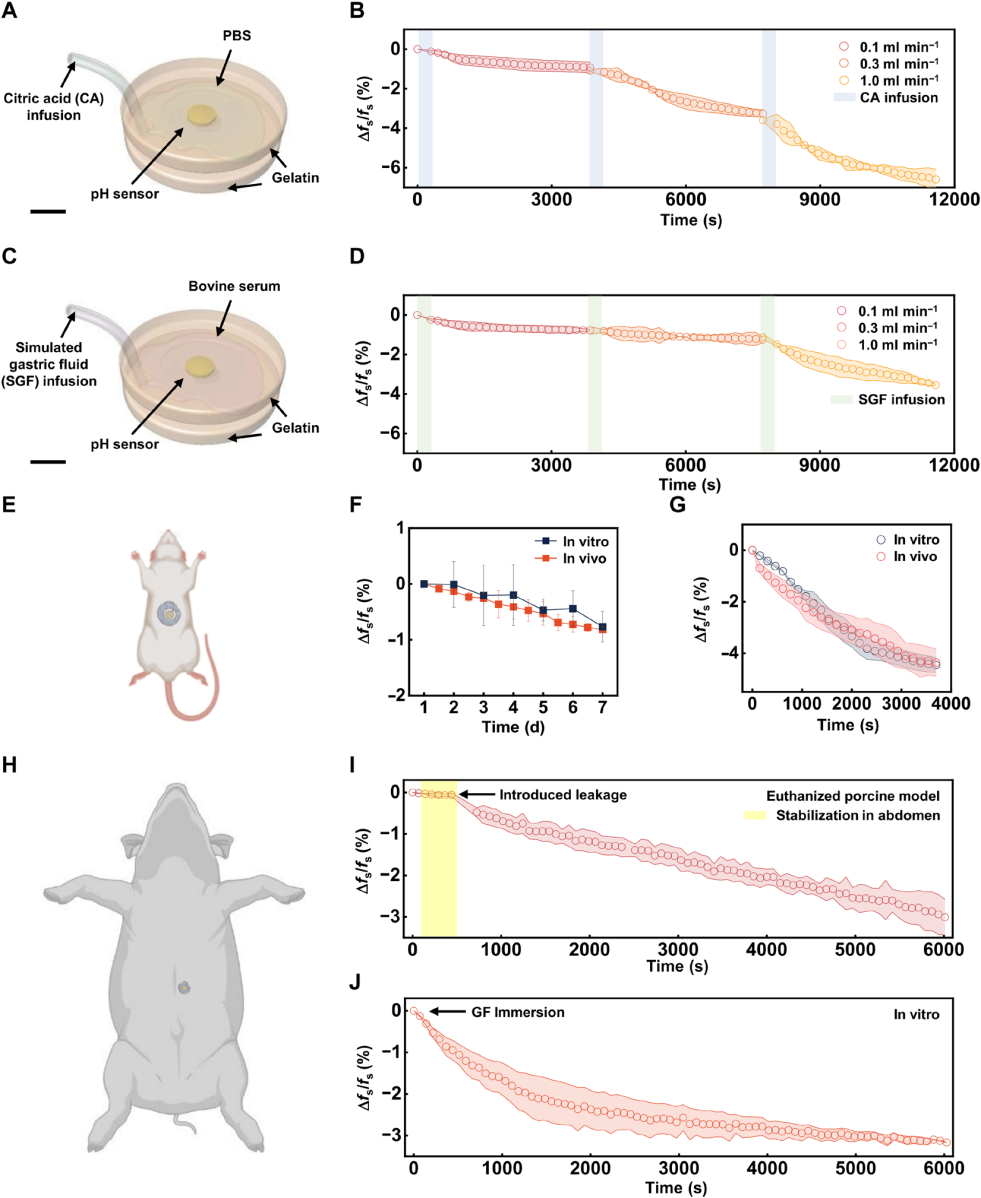
In vitro and in vivo demonstrations of wireless measurements of pH. (**A** and **C**) Schematic illustrations of experimental setups for in vitro wireless pH sensing. This setup mimics gastric leakage in the abdomen, with the pH sensor embedded between gelatin gels (diameter = 60 mm and thickness = 5 mm) and soaked in PBS. A syringe pump (not shown) controls the rate of citric acid (CA)/simulated gastric fluid (SGF) infusion. Scale bars, 10 mm. (**B** and **D**) Continuous measurements of Δ*f*_s_/*f*_s_ after three separate sessions of simulated gastric leakage with increasing leakage rates (0.1, 0.3, 1.0 ml min^−1^). The shaded areas denote the controlled CA/SGF infusion (each of 5 min), separated by 1-hour time intervals. (**E**) Schematic illustration of in vivo pH sensing in the abdomen of a rat model. (**F**) In vivo measurements of Δ*f*_s_/*f*_s_ for pH sensors implanted in rat models for 7 days and in vitro validation. (**G**) Continuous measurements of simulated gastric leakage [with citric buffer (pH = 6)] after the 7-day in vivo implantation and in vitro incubation in PBS. (**H**) Schematic illustration of pH sensing in the abdomen of a euthanized porcine model. (**I**) Continuous measurement of Δ*f*_s_/*f*_s_ for 6000 s, with 5 min of sensor stabilization in the abdomen (by inserting the sensor into an open abdomen region close to the stomach, denoted as the shaded area), followed by an introduced gastric leakage, where a 2-cm incision near the pylorus allows sufficient (~20 to 30 ml) gastric content leakage and full immersion of the sensor. (**J**) Continuous measurement of Δ*f*_s_/*f*_s_ in vitro by immersing the pH sensor in the collected, leaked gastric fluid as in (I).

Evaluations of the performance of the device focus first on characterizations of wireless readout, including the effect of relative height (and different types of medium) between the readout coil and the sensor, as summarized in fig. S15. Sufficient precision (<1% variation in Δ*f*_s_/*f*_s_) can be achieved within a broad range: about 3 cm through air and 2 cm through fat and lean tissues. To further examine the effects of potential lateral and angular misalignments, 3D contour maps (fig. S16) illustrate the values of Re *Z* and Δ*f*_s_/*f*_s_ as a function of angular and lateral misalignment. As shown by the figures, the maximum in Re *Z* can be identified by adjusting the location (within 2.5 cm of the sensor) and angle (with 90°) of the readout coil (fig. S16, A and C), with only small variations in Δ*f*_s_/*f*_s_ (<0.5%; fig. S16, B and D). These results suggest that manual adjustment of the readout coil to maximize the signal will ensure accuracy in the measurement. In this case, the wireless readout scheme has sufficient tolerance to allow for acceptable performance under conditions of minor movements due to flexion of the body, as suitable for intermittent signal checking, even under minor ambulation. In addition, nondestructive medical imaging tools such as ultrasound (e.g., B-mode) may also help determine the exact locations of deployed sensors during regular bedside evaluations (fig. S17). Deep-tissue sensing capabilities and safety issues associated with the radio frequency (RF) exposure is discussed in more details in Discussion and note S3. Additional experiments identify another critical parameter of the sensor, spatial resolution, defined as the maximum distance between the sensor and the site of gastric leakage for which detection is possible. Benchtop experiments reveal that the sensor can readily respond to acid injected at locations up to 6 cm away from the sensor within 1 hour (fig. S18; refer to the caption for details of the experimental setup). Further benchtop characterizations of the device performance involve two different models that mimic the physiological environment of abdomen during gastric leakage (Fig. 4, A and C). The first embeds the sensor between a pair of gelatin gels (from porcine skin, 10 wt % in PBS) with Young’s moduli similar to that of biological tissues [*E* ~ 70 kPa for 10% (w/v) gelatin and *E* = 15 to 100 kPa for skeletal and abdominal wall muscles] (*56*). As shown in Fig. 4A, PBS simulates interstitial fluid and 0.1 M CA (pH = 2.1) simulates gastric fluid. Simulations of leaks include three 5-min sessions of CA infusion at rates of 0.1, 0.3, and 1.0 ml min^−1^, separated by time intervals of ~1 hour. Continuous wireless recording of Δ*f*_s_/*f*_s_ indicates a fast response (within 1000 s) as manifested by a decrease in Δ*f*_s_/*f*_s_ after the injections (Fig. 4B). The magnitude of this decrease increases with and highly depends on the CA injection rate, as expected. The values of pH measured by the sensor after each 1-hour session are 6.7, 6.2, and 5.7. Corresponding measurements with a commercial pH meter are 6.6, 6.0, and 5.4, respectively. In the second setup (Fig. 4C), bovine serum (pH = 7.6) and simulated gastric fluid [SGF; 0.7% (v/v) hydrochloric acid, pH = 1.2] serve as mimics for interstitial fluid and gastric leakage, respectively. This setup exhibits expected trends in Δ*f*_s_/*f*_s_ (Fig. 4D), although with slower decreasing rate compared to that displayed in the first setup, likely attributable to the higher starting pH defined by the serum and the reduced hydrogen ion diffusion due to the higher viscosity of the protein-enriched serum solution. The values of pH measured by the sensor after each 1-hour session are 6.9, 6.6, and 6.1; recorded values from the commercial pH meter are 7.0, 6.4, and 5.9, respectively. The minor discrepancies are primarily caused by the experimental setup: the pH sensors in both cases are sandwiched between gelatin blocks, which may partly inhibit the free expansion of the hydrogel. Further experiments validate the response of the sensor to unspecific acid injection. Studies demonstrate that wireless readouts can be distinguished across multiple sensors involve acquisition of spatiotemporal information from three pH sensors in response to simulated gastric leakage. The results confirm the ability to track the engagement of each sensor with injected acid, even at distant locations, as detectable responses captured in a timely manner less than 1 hour (fig. S19).

Tests of stability and proper operation over the clinically relevant timeframe of ~7 days involve small- and large-animal models (refer to Materials and Methods for more details about animal experiments. With small-animal models (i.e., rats, Fig. 4E), routine, intermittent measurements of resonant frequency shift of devices implanted into their abdominal cavities indicate small variations in Δ*f*_s_/*f*_s_ (<1%) within the 7-day period (Fig. 4F). Such levels of variation correspond to <0.4 unit pH change after 2 hours, sufficiently low to distinguish any acute change in pH due to gastric leakage. The result is consistent with that from in vitro experiments (Fig. 4F), suggesting that the drift in *f*_s_ is mainly due to the gradual intake of water. To facilitate long-term function and stability in vivo, a cage structure made of biodegradable natural wax protects the sensor from potential damage that may be caused by fascia growth and/or unpredictable pressure from surrounding organs, while still allowing fluids to interact with the hydrogel (fig. S20A). Characterization of devices with and without wax cages suggests that the cage does not limit swelling of the hydrogel (fig. S20B), nor does it alter the frequency dependence for inductive coupling (fig. S20, C and D, and table S1). The wax cage design further allows for sutures to enable fixation of the device close to the staple line (fig. S20E), as most gastric leakage occurs close to the proximal third of the greater curvature staple line after sleeve gastrectomy (*57*, *58*). The functionality and reliability of the device after the 7-day stable operation needs to be validated. Figure 4G demonstrates that following the 7-day in vivo implantation or in vitro incubation, the sensors still respond to acute pH changes (pH = 6.0) and yield similar levels of ∆*f*_s_/*f*_s_, comparable to the performance shown in benchtop experiments (e.g., Fig. 2I).

Additional characterization of both Δ*f*_s_/*f*_s_ and *Q* factor immediately after implantation suggests that sensors preswollen in PBS exhibit minimal drift within 30 min (fig. S21), a clear indication that no stabilization or equilibrium time is required following implantation in rat models. Another clinically relevant aspect to consider is that besides suturing, often surgical sites are closed with staples by using endocutters. Studies demonstrate that metal staples do not induce observable variations in Δ*f*_s_/*f*_s_ (fig. S22). Moreover, as LSG often has a surgical site that covers a large area (160 to 300 mm), more than one sensor may be needed to capture all locations of possible leakage. Characterization of multiple sensors in proximity suggests that ∆*f*_s_/*f*_s_ of one device will not be biased by the second (variation <1%), unless they overlap spatially by more than a half in diameter (fig. S23). Last, in the event of a leak, the abdominal cavity may become distended with gas. Characterization of *f*_s_ in multiple devices suggests that the appearance of gas imposes minimal influence on the measurements (fig. S24).

Large-animal experiments conducted using one euthanized porcine model involve the device implanted into the abdominal cavity close to the stomach, with manually initiated leakage of gastric fluid from the animal (Fig. 4, H and I). The result demonstrates a rapid, continuous reduction in Δ*f*_s_/*f*_s_, corresponding to a pH of 6.3 after 6000 s. Related procedures conducted in vitro, namely, measuring the resonant frequency of the sensor after immersion in leaked gastric content for same duration (Fig. 4J), lead to a measured pH as 6.0, consistent with the result obtained with a commercial pH meter (pH = 6.0).

### Assessment of device biocompatibility

**Fig. 5.**
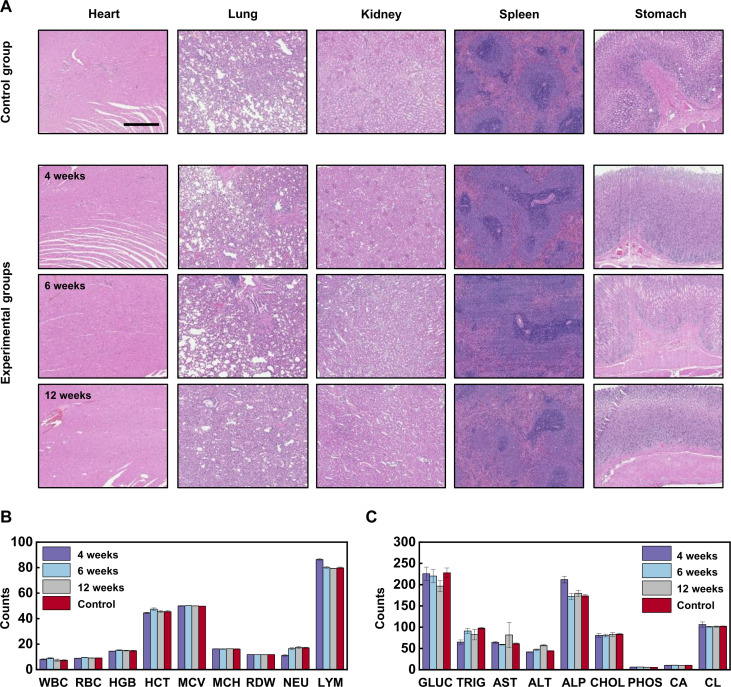
Studies of the biocompatibility studies of the sensor. (**A**) Representative histology images with H&E staining of cross-sectional areas of the heart, lung, kidney, spleen, and stomach of a rat without (control group, first row) and with an implanted device after 4 weeks (experimental group, second row), 6 weeks (experimental group, third row), and 12 weeks (experimental group, fourth row) of implantation (*n* = 3 animals per group over three independent experiments). Scale bar, 500 μm. (**B** and **C**) Analysis of complete blood counts and blood chemistry for rats with and without device implantation (*n* = 3 biologically independent rats). WBC, white blood cell (×1000 μl^−1^); RBC, red blood cell (×1,000,000 μl^−1^); HGB, hemoglobin concentration (g dl^−1^); HCT, hematocrit (%); MCV, mean corpuscular volume (fl); MCH, mean corpuscular hemoglobin (pg); RDW, red blood cell distribution width (%); NEU, neutrophil count (%); LYM, lymphocyte count (%); GLUC, glucose (mg dl^−1^); TRIG, triglycerides (mg dl^−1^); AST, aspartate aminotransferase (U liter^−1^); ALT, alanine aminotransferase (U liter^−1^); ALP, alkaline phosphatase (U liter^−1^); CHOL, cholesterol, total (mg dl^−1^); PHOS, phosphorus (mg dl^−1^); CA, calcium, total (mg dl^−1^); CL, chloride (mEq liter^−1^). In (B) and (C), the results are presented with error bars as means ± SD.

Histological assessments of organ tissues (heart, lung, kidney, spleen, and stomach) near the site of pH sensor deployment indicate no damage to the tissue and no identifiable inflammation responses such as immune cell aggregation associated with implantation after 4, 6, and 12 weeks in rat models (*n* = 3 individual samples), respectively (Fig. 5A). Continuous measurements of body weights of the rat models for 7 days after device implantation show an initial decline, followed by a steady increase, as expected for healthy animals after a major surgery (fig. S25). The results suggest that the implantation does not impose additional stress on the health of the animal nor does it interrupt the normal metabolic processes. Analysis of complete blood counts and blood chemistry also show no signs of systematic inflammation (white blood cell) and organ dysfunction or damage (red blood cell/hemoglobin for bone marrow, aminotransferase for liver, etc.) after 4, 6, and 12 weeks (*n* = 3 individual samples) (Fig. 5B). As the internal environment of the body is in a steady state after postsurgical wound healing (<1 month), these results suggest longer-term biocompatibility. Experimental studies of other PEG-based hydrogels as implants for up to 16-months show minimal inflammatory response and toxicity, as additional support for our claims of longer biosafety (*59*).

## DISCUSSION

This report introduces the materials basis, the fabrication procedures, and the performance characteristics of a wireless, passive, bioresorbable pH sensor designed for early detection of anastomotic leaks after gastric surgery, even before the development of any clinical sequelae. In this system, an acidic environment triggers water and hydrogen ion diffusion into a tough, responsive, and biodegradable PDPAEMA-PEGDA hydrogel, leading to volumetric expansion that, in turn, changes the inductance of an embedding LC circuit. These changes can be measured wirelessly through shifts in the resonant frequency of the circuit, captured through near-field coupling to an external reader. Mechanical simulations reveal the basic aspects of the coupling of the hard and soft materials in these devices, thereby providing important guidelines for design choices that maximize the sensitivity. The response time is ~1 hour, dictated by the kinetics of hydrogen ion diffusion. The timeframe for stable operation is ~7 days both in vivo and in vitro, limited by processes of gradual water permeation and bioresorption. Within this period, the measurement is minimally disturbed by natural movements (e.g., applied force, in-plane uniaxial stretching, out-of-plane bending, and twisting) as well as temperature variations within physiologically relevant range. Compared to previously reported examples of RF resonators for implantable biosensing (*60*, *61*), this technology overcomes challenges in merging wet, hydrated hydrogels with dry, solid-state transient circuit elements, to exploit the attractive features of smart hydrogels (e.g., high ion selectivity, mechanical compliance, biodegradability, etc.), while avoiding adverse effects of surrounding biofluids on the fidelity (e.g., damping and very low *Q* factor) of the LC circuit by appropriately choosing the configuration of the sensor to minimize the parasitic conductive paths in the hydrogel (fig. S26). Use of the device in small- and large-animal models validates its ability to respond quickly and reliably to simulated events of gastric leak. In vivo histological studies, along with assays of blood chemistry and blood count demonstrate the biocompatibility of these sensors. In vitro accelerated tests define the rates of chemical reactions associated with bioresorption. On the basis of previous studies (*62*), other components of the device, including the Zn, W, PVA, and PLGA, all degrade or dissolve in physiological conditions at a much faster rate (<100 days for the slowest metal, Zn). The remaining hydrogel and wax composite degrade at a rather slower rate (up to many months), but they impose very little risk, considering that beeswax (E901) and candelilla wax (E902) are generally recognized as safe by the US Food and Drug Administration (FDA) and authorized in the European Union as a food additives (*63*), and PEGDA-based hydrogels have a long history of FDA approvals for injectables or internal consumption (*64*, *65*). Some major limitations of the current device system involve relatively low sensitivity, nonlinear sensing response, and slow degradation rate, which may hinder potential translational studies and direct applications in clinical medicine. Future improvements and opportunities are in exploiting multifunctionality with other pH-responsive hydrogels [for example, substituting the isopropyl group with ethyl groups yields hydrogels highly sensitive in the range of 7.0 < pH < 7.3 (*66*), suitable for monitoring of anastomotic leaks after small bowel or colorectal surgery] and even other classes of responsive hydrogels (physical, chemical, biological, etc.) for integration into similar, but miniaturized versions of the device reported here, to address other clinical needs in temporary, wireless sensing. The second direction involves extending the wireless sensing range, especially in biological tissues, by either integrating a secondary dipole antenna to the sensor (*67*), or redesigning the LC sensor to impart higher *Q* factor (fig. S27). Another option to circumvent the power and data transfer issues while tolerating minor invasiveness is to explore a combination of tethering and deployable hydrogel device designs (*68*), to allow implantation during surgery and removal like a drain after operation (*69*).

## MATERIALS AND METHODS

### Synthesis of the biodegradable, pH-responsive hydrogel

Mixing PEGDA (*M*_n_ = 700, Sigma-Aldrich) with DPAEMA (Sigma-Aldrich) at a molar ratio of 1:2, along with 2 wt % 2,2-dimethoxy-2-phenylacetophenone (DMPA; Sigma-Aldrich) formed the hydrogel precursor solution. Sonicating for 10 min completely dissolved the DMPA and completed the preparation of the precursor. Ultraviolet (UV) curing of the hydrogel involved infiltrating the precursor into a custom mold (glass slides separated with a spacer) via capillary force and exposing to a 365-nm light source (36 W, CUREbox, Wicked Engineering) for 5 min on each side.

### Fabrication of the pH sensor

Laser cutting (U4, LPKF Laser & Electronics) foils of Zn (Goodfellow) with thickness of 100 μm yielded the inductor with serpentine-shaped spiral coils. Resting the Zn inductor in a polydimethylsiloxane (PDMS) mold (Sylgard 184, Dow; prepared separately via 3D printing and replica molding) and infusing molten bioresorbable wax (a mixture of beeswax and candelilla wax in a weight ratio of 2:1; Sigma-Aldrich) into the channels created a uniform, electrically insulating coating (average thickness about 50 to 100 μm). Demolding the wax-coated Zn coil followed by casting the pH-responsive hydrogel prepolymer between a pair of microscope slides (75 × 50 × 1 mm with a 12-mm-diameter hole drilled in the center) with a 400-μm spacer defined the relative position between the inductor and capacitor, as well as the thickness of the hydrogel. UV curing the hydrogel and removing the excessive parts by cutting with a razor blade defined the pH-responsive, hydrogel-integrated inductor. Laser cutting Zn foil (Goodfellow) with a thickness of 25 μm into 9-mm-diameter discs defined the top and bottom electrodes of the capacitor. Fabrication of the PLGA dielectric layer involved drop casting and slowly drying a solution of PLGA (lactide:glycolide = 65:35, 2 wt % in ethyl acetate, Sigma-Aldrich) on a hydrophobic silicon wafer [passivated by 0.2 vol % trichloro(octadecyl)silane in hexane for 90 s]. Forming the PVA adhesive layer involved spin coating a solution of PVA (*M*_w_ = 13,000 to 23,000, 20 wt % in deionized water, Sigma-Aldrich) over a Zn foil, baking at 70°C on a hotplate for 10 min, and then releasing the film from the foil. Stacking the Zn electrodes, PVA adhesive, and PLGA dielectric layer and then hot pressing at 85°C for 6.5 min formed the capacitor (*C* ~280 to 350 pF). Figure S1 summarizes details associated with the procedures described above. Electrically connecting the inductor and the capacitor with a conductive wax paste [a mixture of tungsten powder (C10, Buffalo Tungsten) and candelilla wax with a weight ratio of 15:1] completed the LC circuits. Casting molten bioresorbable wax into 300-μm-thick discs (diameter = 10 mm) in PDMS molds defined the encapsulation structure. Sandwiching the capacitor between a pair of wax discs and sealing the side (by partially melting the edge of the wax discs using a soldering pencil) completed the fabrication.

### Signal readout and pH measurements

The readout system includes a VNA (E5062A, Agilent Technologies) and a single turn coil. The circular-shaped coil has a diameter of 2.5 cm and an inductance of 70 nH. Setting the VNA to reflective mode enabled measurements of both the real and the imaginary parts of the S-matrix element S_11_. The real part of the impedance across *L*_0_ was derived using the following Eq. 3

$$\text{Re}\;Z=Z_{0}\frac{1-{\left(\text{Re}\;S_{11}\right)}^{2}-{\left(\text{lm}\;S_{11}\right)}^{2}}{{\left(1-\text{Re}\;S_{11}\right)}^{2}+{\left(\text{lm}\;S_{11}\right)}^{2}}$$ (3)

where *Z*_0_ is 50 ohms. Placing the coil over the sensor enabled wireless measurements of the resonant frequency. A customized Visual Basic program installed in the VNA sets the system to continuously record shifts in the resonant frequency. Calibration and verification of pH for all buffer solutions and biofluids are conducted using an ion-sensitive field-effect transistor pH meter (S2K712, ISFETCOM).

### Finite element analysis

Commercial software ABAQUS was used to simulate the expansion of the pH sensor (pH-responsive hydrogel, wax, and Zn inductor) under different pH values and its motion artifacts caused by natural movements (e.g., applied pressure, in-plane uniaxial stretching, out-of-plane bending, and twisting). The hydrogen volume expansion was modeled by thermal expansion, with the thermal expansion α∆*T* to be identical to the square root of the area expansion in Fig. 2D, where α represents the effective coefficient of thermal expansion and *∆T* represents the effectively temperature increase. Eight-node linear reduced integration elements (C3D8R) were applied to Zn inductor, and hybrid elements (C3D8RH) were applied to the hydrogel and wax. The mesh convergence was guaranteed for all cases and the total number of elements was up to 600,000. The elastic modulus (*E*), Poisson’s ratio (ν) are *E*_Zn_ = 108 GPa, ν_Zn_ = 0.25 for zinc and *E*_wax_ = 55 MPa, ν_wax_ = 0.5 for wax. Hydrogel is modeled as a Mooney-Rivlin hyperelastic incompressible material with an initial modulus of 6.5 to 9.8 MPa for different pH values (fig. S14). Previous studies of related hydrogel materials suggest that viscoelastic effects and their dependence on the degree of swelling have a minor influence on the behavior of the empirically calibrated responses of the sensors (*70*). Commercial software Ansys HFSS was used to perform the parameter study for the resonance frequencies and *Q* factors under different pH values and motion artifacts. Deformed Zn inductors simulated by ABAQUS were imported into Ansys HFSS for electromagnetic simulation. Lumped port was used with an impedance according to the matching capacitor (280 pF). An adaptive mesh was adopted to ensure computational accuracy. The electromagnetic parameters in the material library of Ansys HFSS and fig. S23 were used in the simulation.

### Implantation of the bioresorbable pH sensor into the abdominal cavity of live and euthanized animals

All procedures associated with the animal studies followed the recommendations in the Guide for the Care and Use of Laboratory Animals of the National Institutes of Health. The Institutional Animal Care and Use Committee of Washington University in St. Louis approved the protocol (protocol no. 22-0022). Adult male Lewis rats (weight ~330 to 360 g; 14 to 16 weeks old; purchased from Charles River Laboratories, Wilmington, MA) were acclimatized up to 7 days before the surgery. All rats were housed in a central animal care facility with food (PicoLab rodent diet 20, Purina Mills Nutrition International, St. Louis, MO) and water ad libitum. The rats were under general anesthesia with inhaled isoflurane vapor (4% for induction and 2% for maintenance) during the implantation surgery. All rats were monitored for signs of infection and distress postoperatively.

The surgery procedures on living rats started with appropriate anesthesia, preparation, and sterilization with betadine and isopropanol solutions. The rats were placed in the supine position and performed via laparotomy with a 3-cm midline incision on the abdomen. The stomach was found with an atraumatic forceps. The UV radiation–sterilized bioresorbable pH sensor was first swollen in PBS for 2 hours before inserted into the abdomen close to the pylorus. The abdomen muscle and skin were closed with 5-O Vicryl and 4-O sutures, respectively. A photographic presentation of the surgery and device implantation procedure was illustrated in fig. S28.

Functional tests were performed on a euthanized porcine model and multiple rat models. The pig (weight ~45 to 55 kg; Oak Hill Genetics, LLC, IL) was placed in the supine position and performed via laparotomy with a 20-cm midline incision on the abdomen. The stomach was exposed and held with an atraumatic forceps, and a 2-cm incision was made in the stomach wall near pylorus to allow gastric content leakage. The pH sensor (PBS swollen for 2 hours) was inserted into the abdomen, close to the pylorus incision. The readout coil resides directly above the sensor to optimize the signal strength, for 2 hours of continuous recording of the resonant frequency. During the experiment, the abdomen was left open as a result of limited detection range of wireless readout coil of VNA. After the 7-day stability test, the rats were placed in the supine position under anesthesia. Four milliliters of citric buffer solutions (pH = 6.0) injected into the abdomen of the rat models simulates the acidic environment after gastric leakage. The readout coil was positioned directed above the sensor and underwent 1-hour continuous recording of resonant frequency.

### Evaluation of histology, hematology, and blood chemistry of rats

All procedures followed protocols approved by the Institutional Animal Care and Use Committee of Washington University in St. Louis (protocol no. 22-0022). UV radiation was used to sterilize the bioresorbable pH sensors before implantation. The implantation procedures were as described above without stomach incision. Daily checking, weighing, and care of the rats ensured their normal health conditions and stress levels. Collection of blood and explantation of organs (heart, lung, kidney, spleen, and stomach) happened at 4-, 6-, and 12-week postimplantation. Blood samples were collected from jugular vein with 26-gauge syringe. The blood samples for complete blood counts were stored in K-EDTA tubes, shook well, and kept with ice. Blood samples for chemistry tests were stored in serum separator tubes and spun entirely to separates serum from clotted red cells. The serum layers were transferred to another empty tube and kept with dry ice. Charles River Laboratories conducted the complete blood counts and blood chemistry tests. The explanted organs were stored in 10% neutral-buffered formalin in 50-ml conical tubes in preparation for histology studies. Mouse Histology and Phenotyping Laboratory at Northwestern University conducted paraffin embedding, sectioning (section thickness = 4 μm, one to two tissue cuts on each slide), and hematoxylin and eosin (H&E) staining based on standard protocol. Pathology Core Facility at Northwestern University performed the slide-based assays of tissue samples.

### Statistical analysis

All analyses were quantitatively performed with at least three samples per group or per test. Numerical data were converted to and presented as means SD whenever applicable.

## Supplementary Materials

### This PDF file includes:

Supplementary Text Figs. S1 to S30 Table S1 References
