# Supplementary material for: Bioresorbable, wireless, passive sensors for continuous pH measurements and early detection of gastric leakage
Source: Sci Adv. 2024 Apr 19;10(16):eadj0268. doi: 10.1126/sciadv.adj0268 (PMC11029800; doi:10.1126/sciadv.adj0268)
Supplement: Supplementary file 1 — Supplementary Text Figs. S1 to S30 Table S1 References [file sciadv.adj0268_sm.pdf]

Science Advances
AAAS

Supplementary Materials for

Bioresorbable, wireless, passive sensors for continuous pH measurements and early detection of gastric leakage

Shuo Li *et al.*

Corresponding author: Di Lu, dilu@ustc.edu.cn; John A. Rogers, jrogers@northwestern.edu

Sci. Adv. **10**, eadj0268 (2024)
DOI: 10.1126/sciadv.adj0268

This PDF file includes:

Supplementary Text
Figs. S1 to S30
Table S1
References

Supplementary Text

Supplementary Note 1. Diffusion model for pH-responsive hydrogel. A diffusion model was established to study the diffusion of hydrogen ions in the pH-responsive hydrogel. Because the thickness of the sample (*h*) is much smaller than its length and width, the model was simplified to 1D diffusion model. With an initial concentration c_0 and surrounding concentration c_s of hydrogen ions, the governing equation and initial and boundary conditions are

$$\begin{cases} \frac{\partial c}{\partial t} = D\frac{\partial^2 c}{\partial x^2} & x \in [0, h/2] \\ \frac{\partial c}{\partial x} = 0 & x = 0 \\ c = c_s & x = h/2 \\ c = c_0 & t = 0 \end{cases} \quad \text{(S1)}$$

where *x* is the direction along the thickness and *D* is the diffusivity of hydrogen ions in the hydrogel. The concentration at the middle surface of the hydrogel (c_m) is derived as below:

$$c_m = \frac{4(c_0 - c_s)}{\pi}\sum_{n=0}^{\infty}\left[\frac{(-1)^n}{2n+1}e^{-\frac{D(2n+1)^2\pi^2}{h^2}t}\right] + c_s \quad \text{(S2)}$$

h is 0.4 mm and*D* is in the order of 1×10^{-6}-1×10^{-5} $mm^2\ s^{-1}$,(*47*) which yields characteristic diffusion time to be 4,000-40,000 s to reach equilibrium (reach ~ 90% concentration c_s).

Supplementary Note 2. Effective modulus of the Zn serpentine structure. According to literature,(*51*) the component of the flexibility matrix for axial force and displacement is

$$c = \frac{\lambda^3 n}{16Eb^3h}\left[4\left(\frac{2H}{\lambda}-1\right)^3 + 6\pi\left(\frac{2H}{\lambda}-1\right)^2 + 24\left(\frac{2H}{\lambda}-1\right) + 3\pi\right] \quad \text{(S3)}$$

where λ is the period length of each cell, *n* is the total cell number, *H* is the height of the serpentine, *E* is the material modulus, *b* is the width and *h* is the thickness (Supplementary Fig. S13). The effective modulus of the serpentine is given by

$$E_{eff} = \frac{L_{eff}}{cS_{eff}} = \frac{n\lambda}{chH} \quad \text{(S4)}$$

where L_{eff} is the effective total length and S_{eff} is the effective cross-sectional area.
With restrictions of width-thickness ratio (≥ 1), total length (< 0.3 m) and total area coverage (diameter < 2.5 cm), the effective modulus can be reduced to 3.9 MPa with the parameters E_{Zn} = 108 GPa, $b = h = 0.1$ mm, $H = 2$ mm, $\lambda = 2.1$ mm.

Supplementary Note 3: Wireless signal detection range and radio frequency (RF) safety. A power of 1 mW (0 dBm) from the vector network analyzer (VNA) enabled wireless readout for the results presented in the main part of the paper, which yields an effective sensing range of 2 cm through biological tissues (as shown in Supplementary Fig. S15e,h). For application over larger distances, such as those that might be needed for obese patients, the design of the readout coil can be modified. As shown in a specific example in Supplementary Fig. S30a, a square loop spiral coil with 7 turns and total width of 102 mm increases the signal detection range, with capacitance and inductance values selected to provide a high Q-factor (Q > 20) at the resonant frequency of interest (f_s ~ 15-30 MHz). Lumped ports were used to obtain the *S*-parameter that describes the input-output

relationship between the transmission and receiver coil and gives the efficiency for signal transmission. As shown in Supplementary Fig. S30b, the experimental noise level (as defined by the ratio of noise to signal strength) was measured using the VNA and extrapolated to a broader range of output power (0.01 mW-10 W). The results show that the noise decreases by 3 times when output power increases by 10 times and the noise is assumed to be independent of Z distance. The signal transmission efficiency between the transmission coil and the implant was modeled by calculating the magnitude of the S_{21} parameter as $\eta = |S_{21}|^2 \cdot 100$.(*71*) The simulation results reveal that the improved readout coil significantly increases the transmission efficiencies. With 10 W power and 20 cm separation, the readout coil can still capture signals that are significantly larger than the noise (0.0014% > ~ 0.00062%), indicating the ability to detect signal over 20 cm, suitable for obese patients (Supplementary Fig. S30c).

To further validate long-range sensibility with conventional VNA, we built a non-bioresorbable LC sensor with high Q-factor (Q > 100). The device is similar to our pH sensor in overall dimensions (2.5 cm in diameter), but it exploits a thicker wire for the inductive coil (0.5 mm in diameter) and a lower resistance solder joint to connect the inductor and capacitor. Each modification decreases the resistance of the LC circuit by a factor of ~ 3-20, thus leading to a significant enhancement in Q-factor. As shown in Supplementary Fig. S27, the device enables sensing at a distance (*H*) of over 15 cm in air, with a single loop readout coil (20 cm in diameter) when operated with a high power VNA (10 dBm, or 10 mW). The same sensor further enables wireless readout through 15 cm of biological tissue (ground meat). Therefore, long-range sensing is promising for a partially transient or even fully transient system using a conventional VNA readout system.

For radio frequency (RF) safety, the specific absorption rate (SAR) was calculated. Federal Communications Commission (FCC) regulates a maximum SAR level of 1.6 watts per kilogram of body tissue for wireless devices.(*72*) Our simulation results suggest that 1 mW of output power from the VNA corresponds to ~ 10 mW kg^{-1} of maximum SAR (i.e. when the readout coil is directly attached to the skin), well below the FCC limit (Supplementary Fig. S30d). With the improved coil design and 10 W output power, the simulated maximum SAR is 1.4 W kg^{-1} (Supplementary Fig. S30e), also within the range of allowable RF exposure, which guarantees the safety of the device for long-range wireless measurement. Moving the readout coil slightly away (~ 5-10 mm) from the skin will further lower the SAR, without sacrificing the detection range, given that the electromagnetic dissipation in air is much less compared to that in biological tissues.

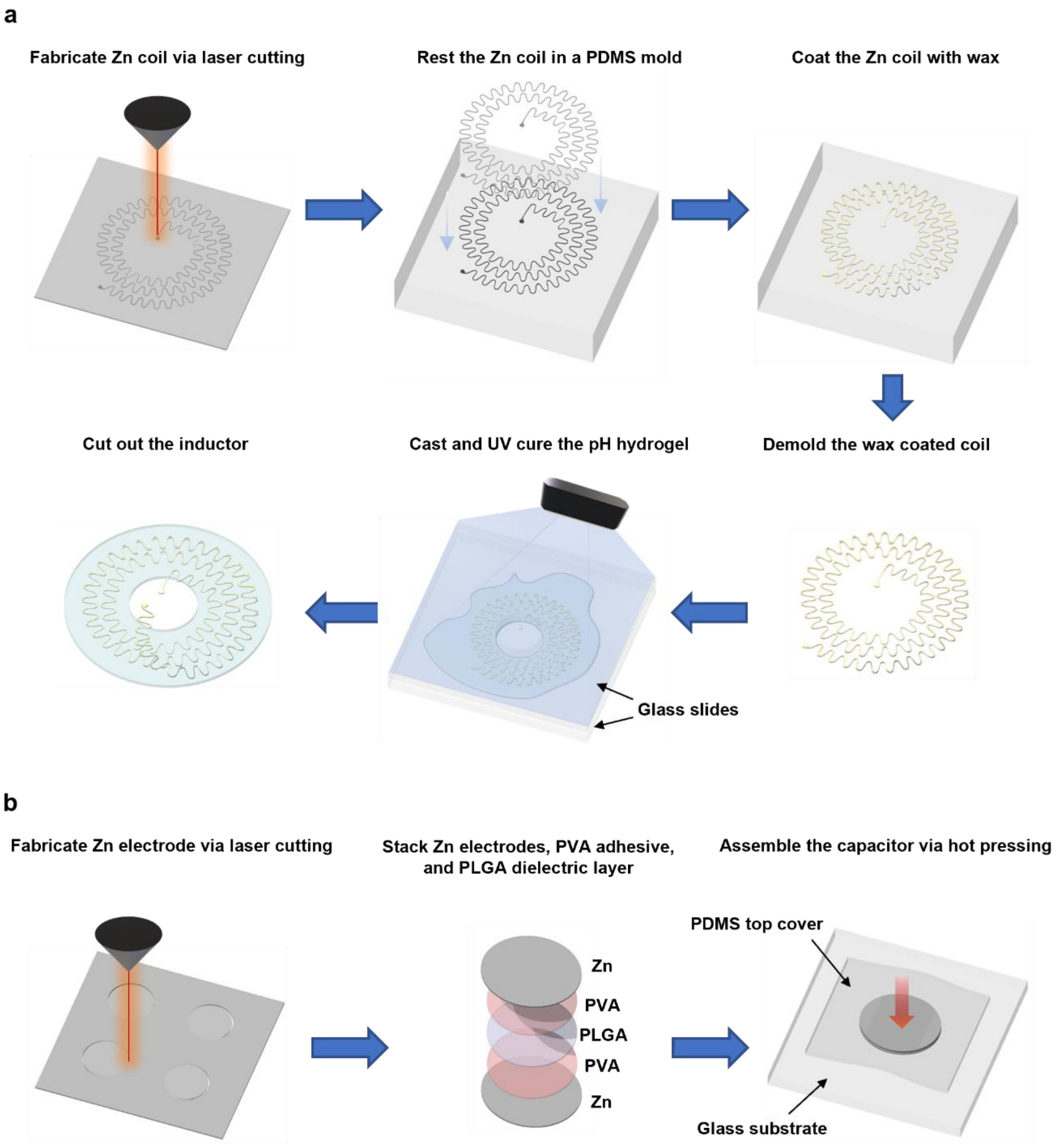

Fig. S1. Schematic illustrations of the procedures for fabricating a bioresorbable pH sensor. (a) Fabrication of the inductor encased in a pH-responsive hydrogel. (b) Fabrication of the bioresorbable capacitor (before wax encapsulation).

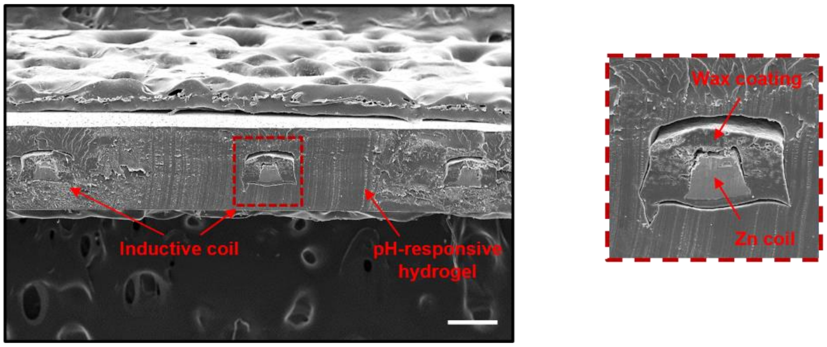

Fig. S2. Hydrogel-encased wax-coated Zn inductor

Cross-sectional scanning electron microscope (SEM) images of a wax-coated Zn inductor cast in a pH-responsive hydrogel (80° tilted view). Inset: magnified view of the Zn coil and the wax coating. Scale bar = 200 µm.

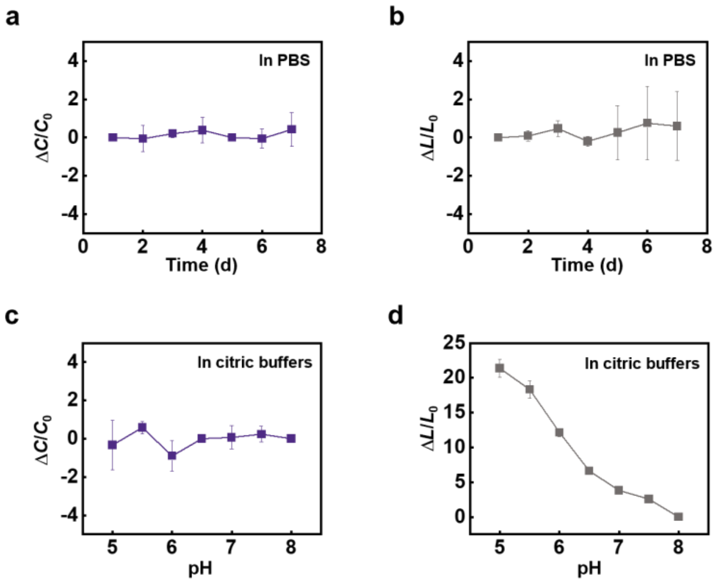

Fig. S3. Characterizations of capacitance and inductance of components in the sensor.
(a) Variation in capacitance (C) of the wax encapsulated capacitor in PBS for 7 days. (b) Variation in inductance (L) of the pH-responsive hydrogel encased inductor in PBS for 7 days. (c) Variation in C in different pH buffers in the range of 5.0-8.0 after 2 h immersion. (d) Variation in L in different pH buffers in the range of 5.0-8.0 after 2 h immersion.

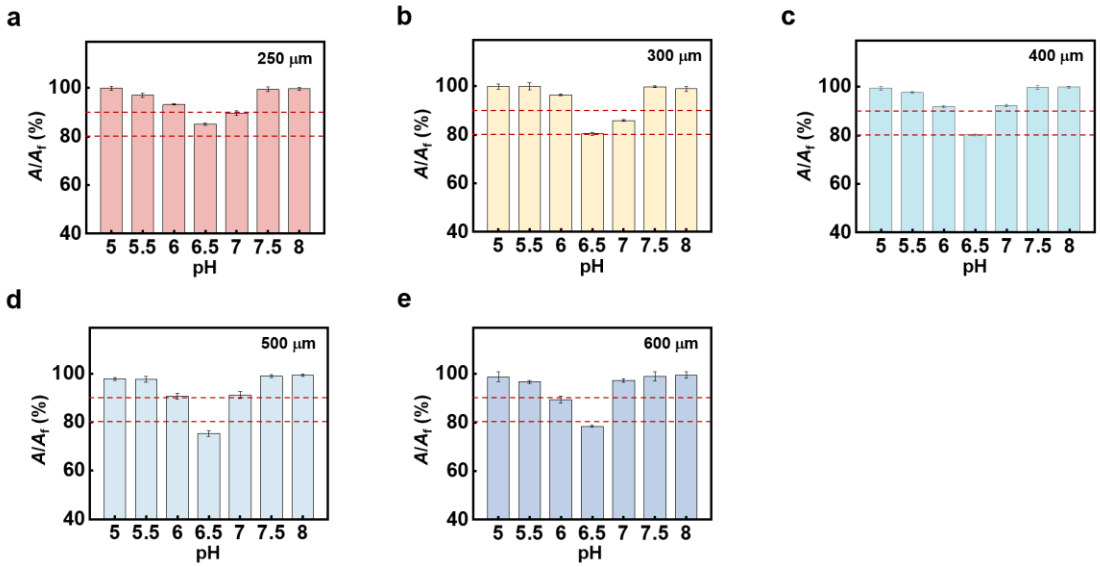

Fig. S4. Optimization of hydrogel thickness.

(a)-(e). Thickness dependent (from 250 μm to 600 μm) area expansion of the pH-responsive hydrogel as a function of pH in the range of 5.0-8,0. Histograms show the percentage of the area of the hydrogel (A) measured at 2 h compared to that at 24 h (A_f) after immersion in citric buffer solutions. Only 400 μm hydrogels show A/A_f above 80% across all pH.

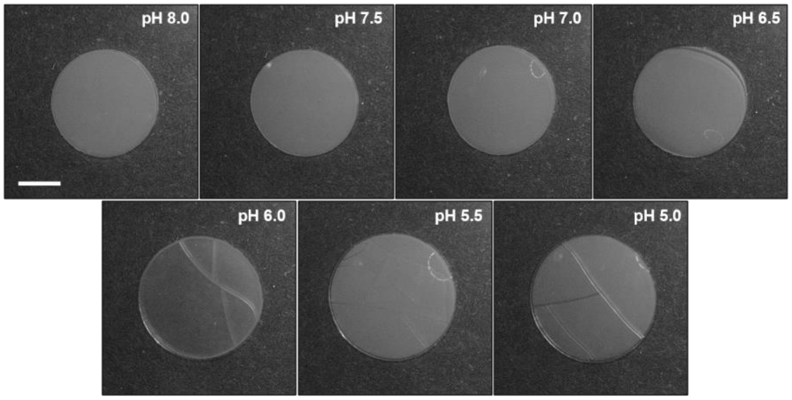

Fig. S5. pH-responsive hydrogels immersed in buffer solutions of different pH.

Photographs of the pH-responsive hydrogels after 2 h of immersion in citrate buffer solutions of different pH. The images show cracks formation in pH 6.0-pH 5.0. Scale bar = 5 mm.

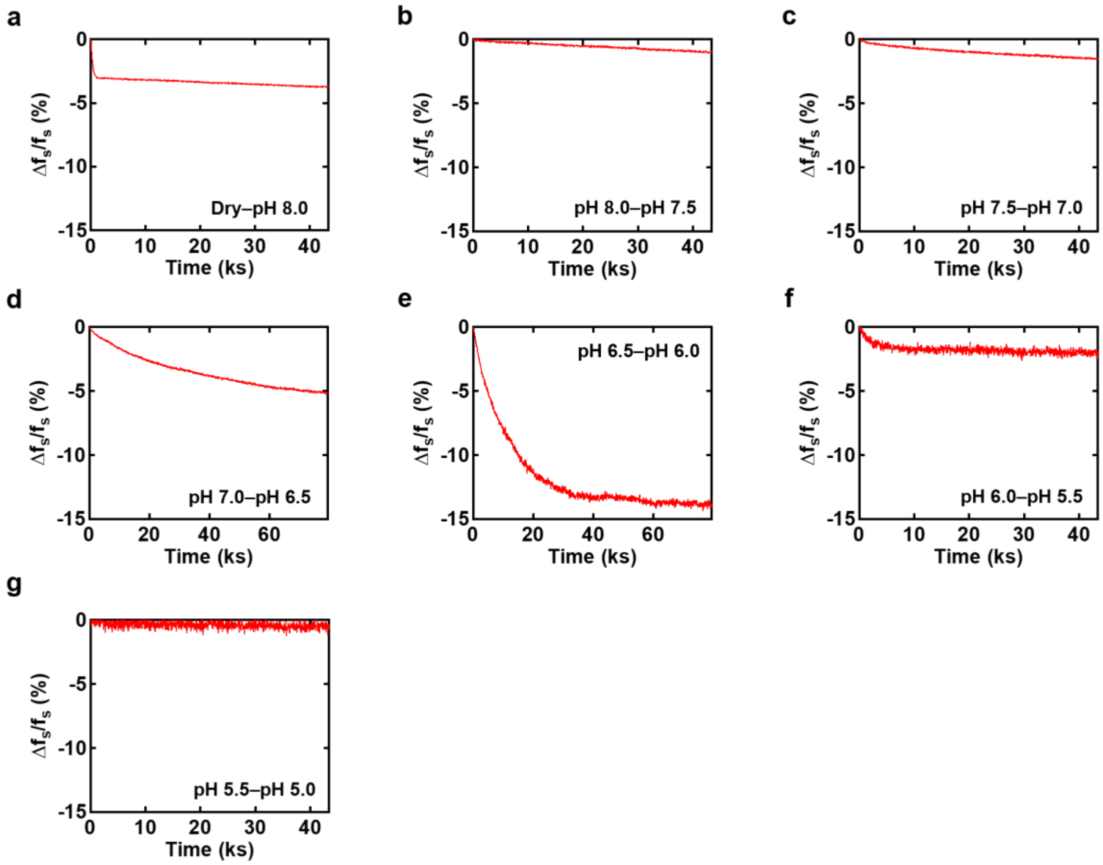

Fig. S6. Prolonged monitoring of the variation in resonant frequency as a function of time. (a-g) Continuous measurements of $\Delta f_S/f_S$ of the same device were conducted from the dry state to the swollen state in pH buffer solutions with pH from 8.0 to 5.0 with an interval of 0.5. Over consecutive periods of 12 h, the sensor was first immersed in buffer solutions of higher pH, then collected by removing the residue buffer solutions, before being immersed in the new buffer solution with lower pH. The results verify the long equilibrium time (4,000-40,000 s) under each state, limited by the diffusivity of hydrogen ions (1×10^{-6}-1×10^{-5} mm^2 s^{-1}).

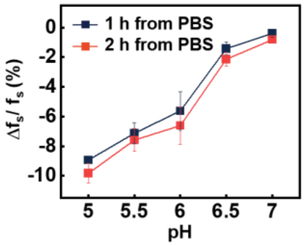

Fig. S7. Time dependent sensor response in citric buffer solutions.

Extrapolated $\Delta f_s/f_s$ as a function of pH after 1 h and 2 h in citric buffer solutions after fully swollen in PBS.

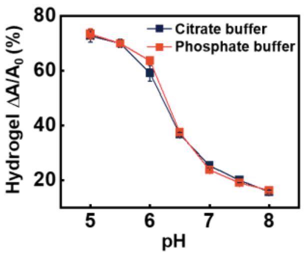

Fig. S8. Comparison of hydrogel swelling behavior in two types of buffer solutions.

Measurements of the area expansion of the pH-responsive hydrogel after 2 h of immersion in citrate (citric acid-trisodium citrate, 0.1 M) and phosphate buffers (monopotassium phosphate dipotassium phosphate, 0.1 M) show they are nearly identical, suggesting that the pH-triggered swelling behavior is independent of buffer type.

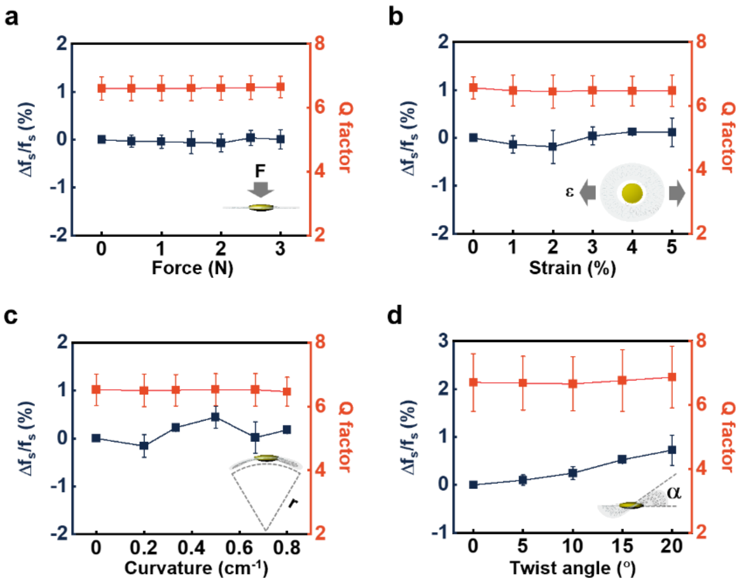

Fig. S9. Effects of motion (experiment results).

Measured $\Delta f_s/f_s$ and Q-factor as a function of (a) applied force, (b) axial strain, (c) bending curvature, and (d) twisting. The results indicate no significant change with these parameters. Insets: schematic illustrations of the device under these same deformations, characteristic of those that may occur during natural movements of the animal.

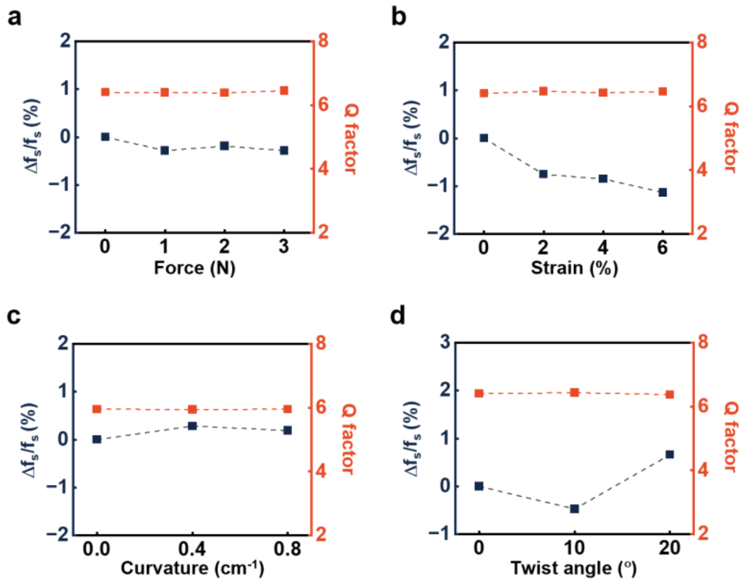

Fig. S10. Effects of motion (simulation results).

Simulated $\Delta f_s/f_s$ and Q-factor as a function of (a) applied force, (b) in-plane uniaxial stretching, (c) out-of-plane bending curvature, and (d) twisting.

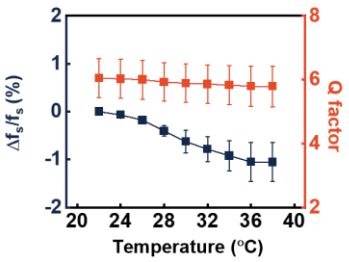

Fig. S11. Effect of Temperature.

Measured $\Delta f_s/f_s$ and Q-factor as a function of environmental temperature. The sensor was stabilized at each temperature for at least 2 min before the data collection.

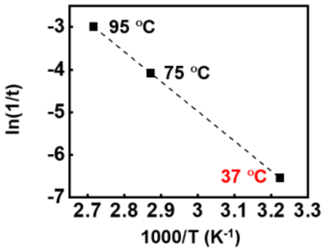

Fig. S12. Dissolution kinetics of the pH-responsive hydrogel.

Time of dissolution in days (derived from Fig. 2k) plotted on a logarithmic scale as a function of temperature (1000/K) for the pH-responsive hydrogel. A fitted linear relationship yields extrapolated times for 37 °C.

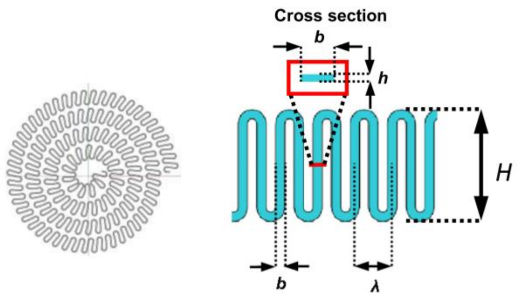

Fig. S13. Schematic illustrations of a serpentine structure used to define the parameters for simulations of the mechanical properties of the Zn serpentine inductor.

Left: overall structure. Right: magnified view of the serpentine, where *h* is the thickness, *b* is the width, *λ* is the unit cell length, and *H* is the unit cell height.

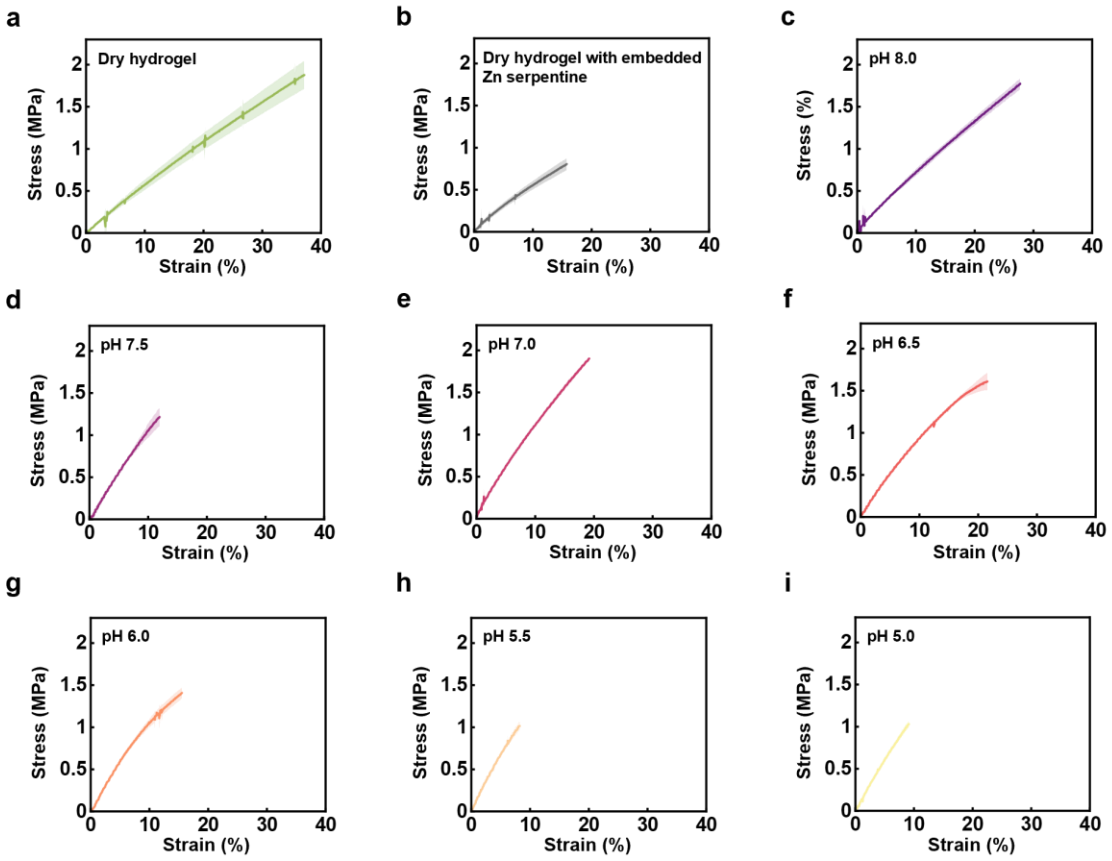

Fig. S14. Mechanical Properties of the pH-responsive hydrogel.

(a)-(i) Stress-strain curves of the dry pH-responsive hydrogel, dry hydrogel with embedded Zn serpentine structure, and hydrogels swollen in citric buffer solutions of different pH from 8.0 to 5.0 with an interval of 0.5. The shaded area represents measurements of at least 3 specimens.

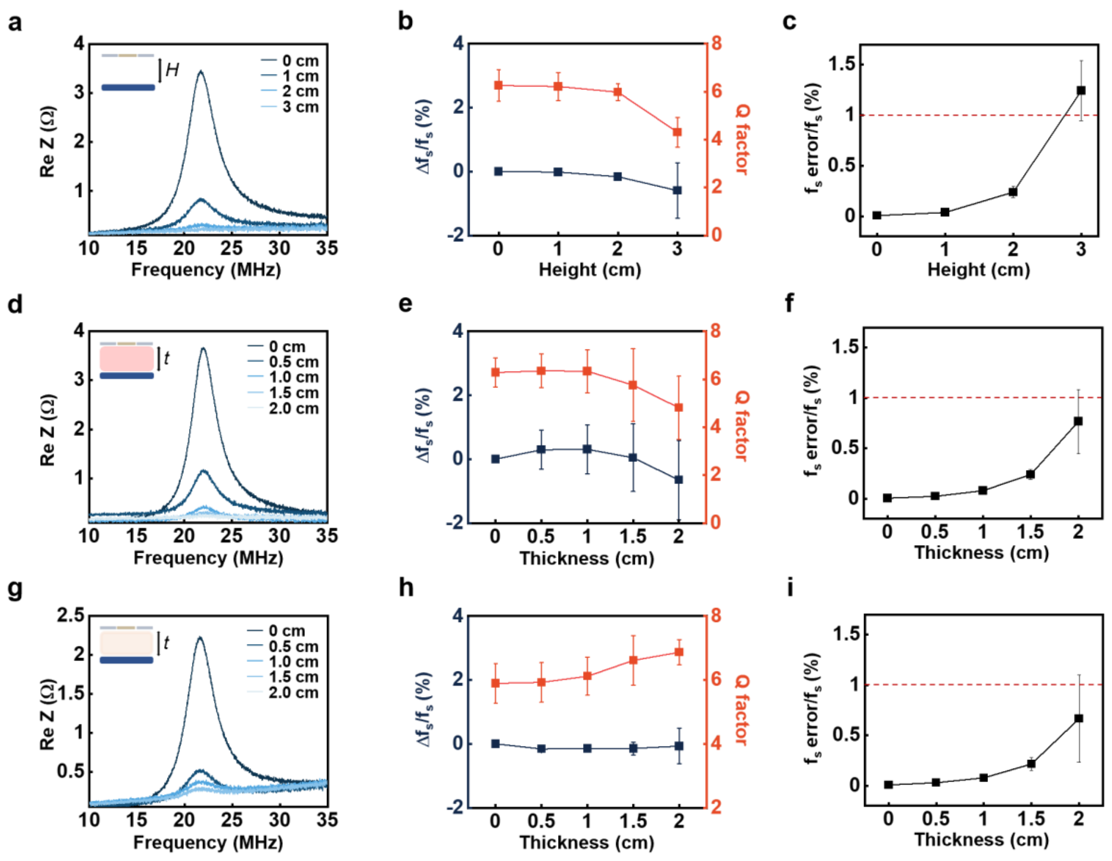

Fig. S15. Characterizations of wireless readout performance.

(a) Measured Re *Z* as a function of frequency for sensors placed over the readout coil with different heights (in air). (b) Measured $\Delta f_s/f_s$ and Q-factor as a function of height. (c) Amplitude of error ($f_{s\ error}/f_s$) as a function of height. d-f. Measurements of Re Z, $\Delta f_s/f_s$, and Q-factor, and amplitude of error for sensors placed on a readout coil through different thicknesses of lean tissue (chicken breast). g-i. Measurements of Re Z, $\Delta f_s/f_s$, and Q-factor, and amplitude of error for sensors placed on a readout coil through different thicknesses of fat tissue (park belly).

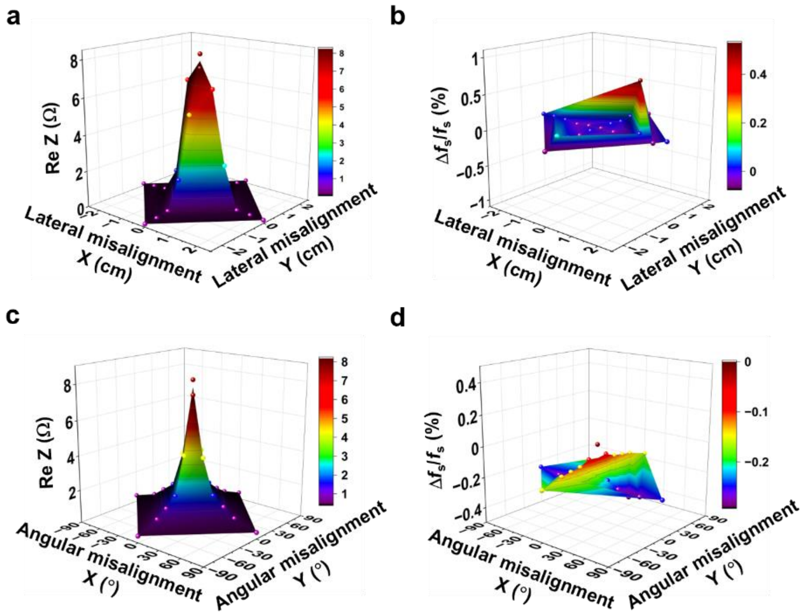

Fig. S16. 3D contour maps showing wireless readout signal strength.

Signal strength (defined by the magnitude of Re Z) as a function of (a) lateral misalignment and (c) angular misalignment. 3D contour maps showing resonant frequency shifts ($\Delta f_s/f_s$) as a function of (b) lateral misalignment and (d) angular misalignment.

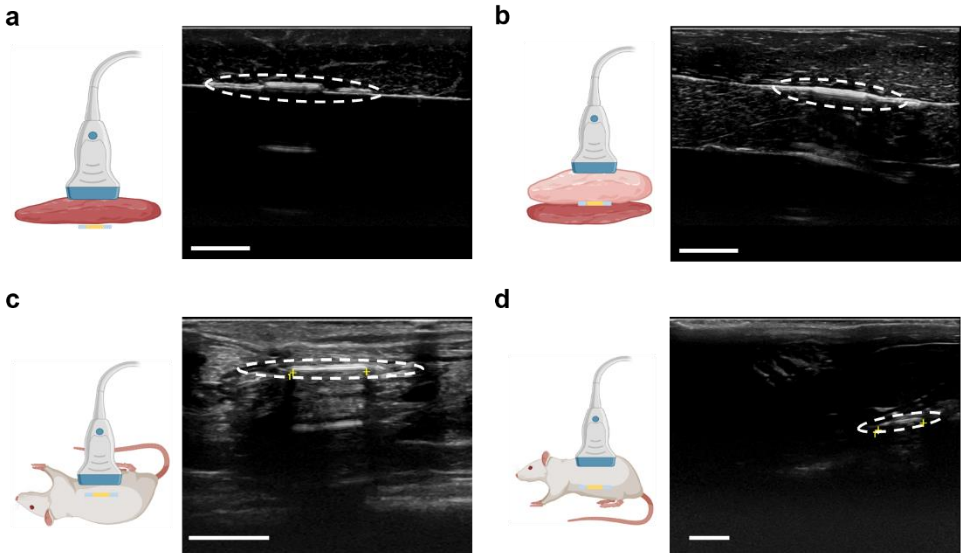

Fig. S17. Ultrasound imaging of pH sensors in vitro and in vivo.

Schematic illustration of the experimental setups (left) and B-mode images (right) of devices embedded (a) in 1 cm porcine tissue. (b) in 3 cm chicken tissue. (c) in the abdomen of a living rat, imaged in supine position. (d) in the abdomen of a living rat, imaged in prone position. Scale bars = 1 cm.

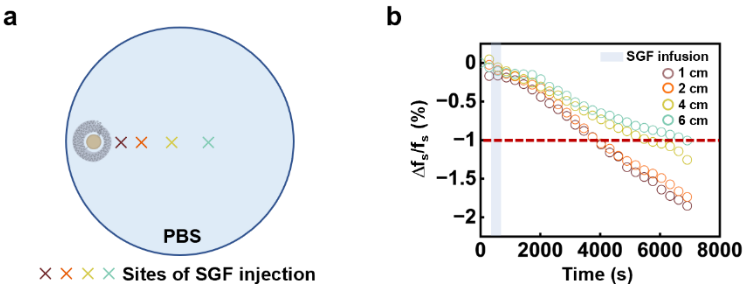

Fig. S18. Spatial resolution of the pH sensor.

(a) Schematic illustration of the experimental setup. (b) Measurements of $\Delta f_s/f_s$ as a function of time for different distances between the sensor and perforation site. A pool of PBS (~ 20 cm in diameter, ~ 1 cm in depth) serves as the basis for simulating the chemical environment of the sensor (an internal environment filled with interstitial fluids). The experiment involves careful injection of 4 mL of simulated gastric fluid (SGF) (to avoid macroscopic fluid flow), 5 min after the sensor reaches equilibrium in PBS and then recording continuously for 2 h. $\Delta f_s/f_s$ = -1% is set as threshold.

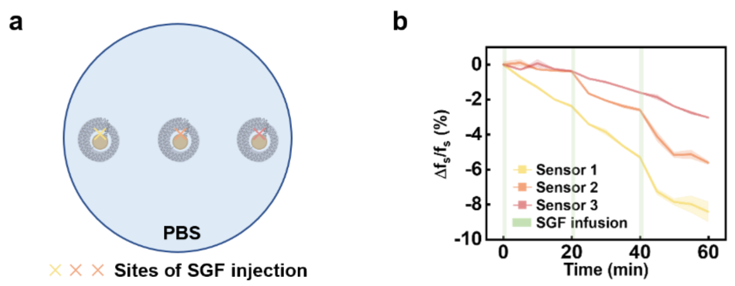

Fig. S19. Spatiotemporal mapping of multiple pH sensors in response to simulated gastric leakage.

(a) Schematic illustration of the experimental setup. (b) Measurements of $\Delta f_s/f_s$ as a function of time for 1 h of 3 pH sensors with different f_s baselines. 2 mL of simulated gastric fluid (SFG) is injected at time 0, 20 min, and 40 min, directly over sensor 1, 2, and 3, respectively.

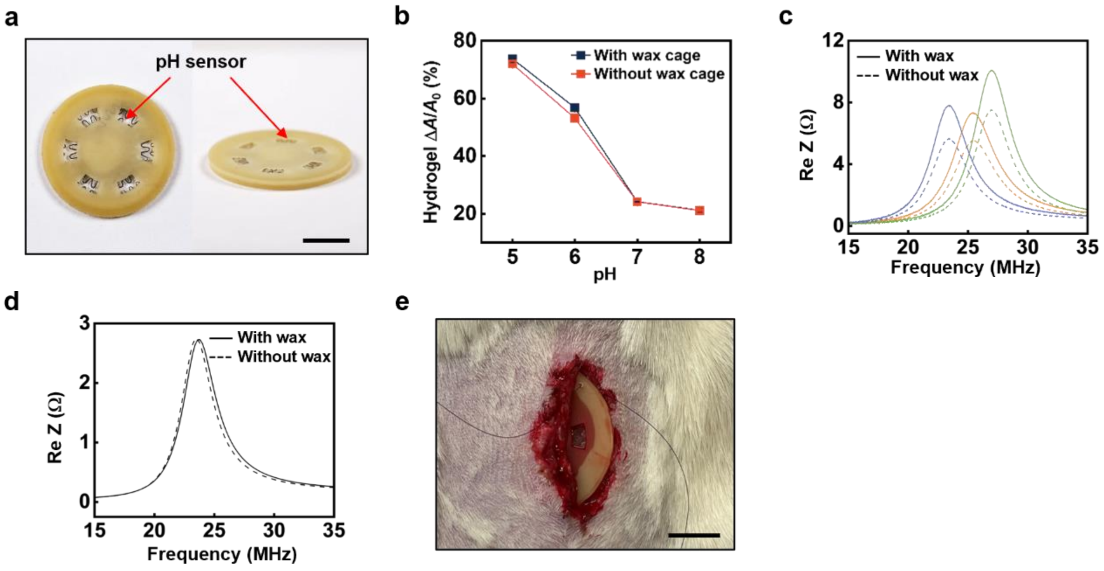

Fig. S20. Biodegradable natural wax cage.

(a) Photograph (both top and side view) of a biodegradable wax cage structure used to protect the pH sensor from damage in vivo after device implantation. (b) Measurements of the area expansion ($\Delta A/A_0$) of the pH-responsive hydrogel as a function of pH after 2 h of immersion with and without the wax cage. (c) Measured Re Z as a function of frequency for 3 sensors of different fs with and without wax cages. (d) Simulated results for Re Z as a function of frequency for a pH sensor with and without a wax cage. (e) Photograph of device implantation and its fixation to rat's abdominal wall with a suture through the wax cage. Scale bars = 1 cm.

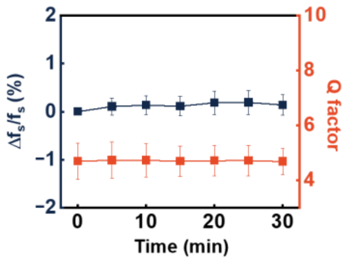

Fig. S21. Short-term stability of sensor response in rat models.

Continuous measurements of $\Delta f_s/f_s$ and Q-factor for 30 min directly after sensor implantation in rat models.

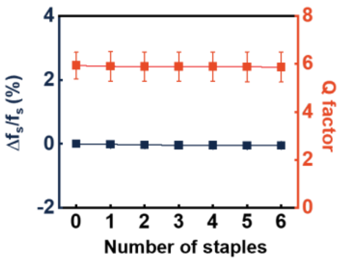

Fig. S22. Sensor response under the perturbation of medical staples.
Measured $\Delta f_s/f_s$ and Q-factor as a function of number of staples positioned near the sensor.

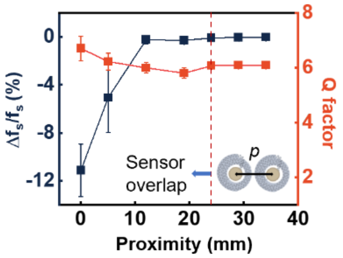

Fig. S23. Sensor response under the perturbation of other sensors.
Measured $\Delta f_s/f_s$ and Q-factor of a pH sensor as a function of its proximity to another.

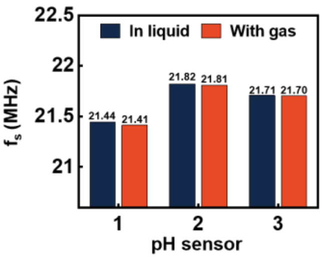

Fig. S24. Resonant frequency as functions of transmission media.

Comparison of resonant frequency measured using 3 different pH sensors via transmission media of liquid and liquid distended with gas, respectively.

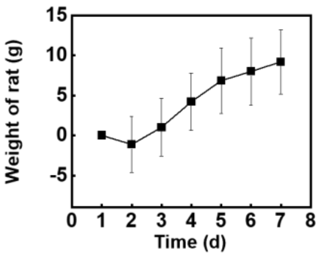

Fig. S25. Characterization of rat’s body weight.
Changes in body weights of rats after implantation of the device.

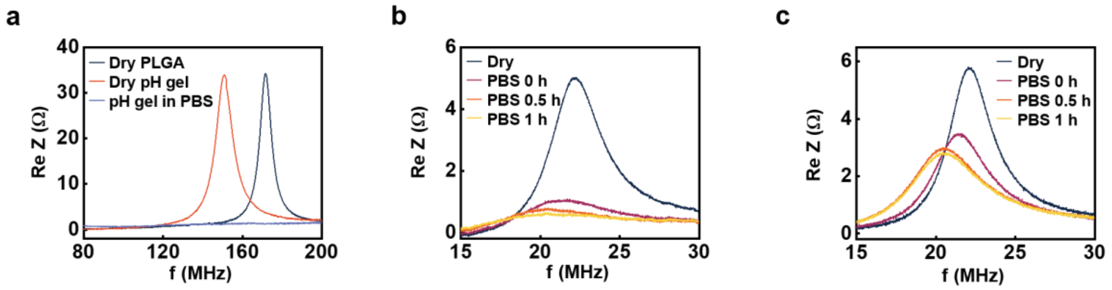

Fig. S26. RF responses of pH sensors with alternative designs.

(a) Measured Re *Z* as a function of frequency for a sensor design with a PLGA dielectric (used in this study), compared to a design with a pH-responsive hydrogel dielectric (a common strategy applied by many other hydrogel integrated passive LC biosensors). The curves clearly show that the pH-responsive hydrogel cannot be used as a dielectric layer, because the PBS-swollen hydrogel will electrically short the plates of the capacitor, thereby eliminating the LC-resonant peak. (b) Measured Re *Z* as a function of frequency for a design that does not involve an insulating wax coating over the Zn inductor. Reductions in the magnitude of the LC-resonance peak suggest resistive losses over time in the inductor until the peak disappears, illustrating the necessity for an insulating coating. (c) Measured Re *Z* as a function of frequency for a design that includes an insulating wax coating over the Zn inductor. Partial reduction in the signal magnitude during the first 0.5 h plausibly results from the electromagnetic dissipation induced by the ionic conductivity of the PBS swollen hydrogel. The fact that the shape of the peak no longer changes after 0.5 h is attributable to the insulating property of the thin layer of wax (thickness = 50-100 μm) over the inductive coil, which can effectively prevent short circuit in the LC sensor.

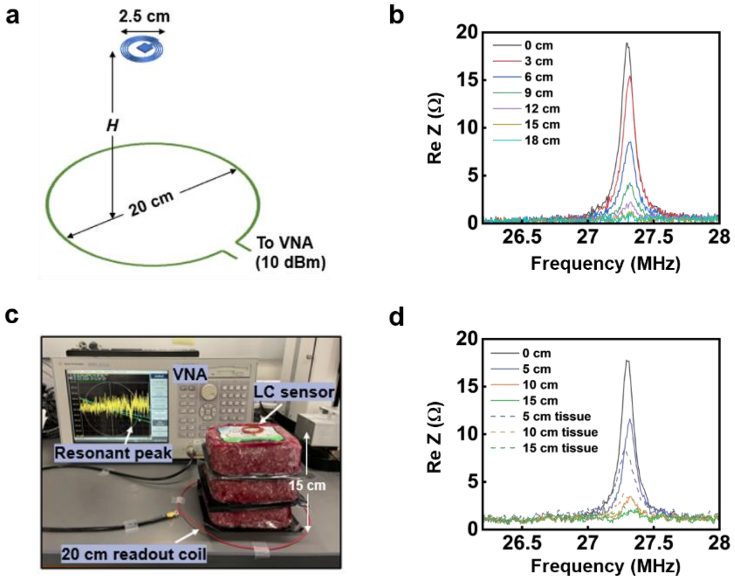

Fig. S27. Long-range wireless readout with a non-transient LC sensor.

(a) Schematic illustration of experimental setup in air. (b) Measured Re Z as a function of frequency in air. (c) Photograph showing the wireless readout through biological tissue (ground meat) using a high power VNA. (d) Measured Re Z as a function of frequency in air and through tissue.

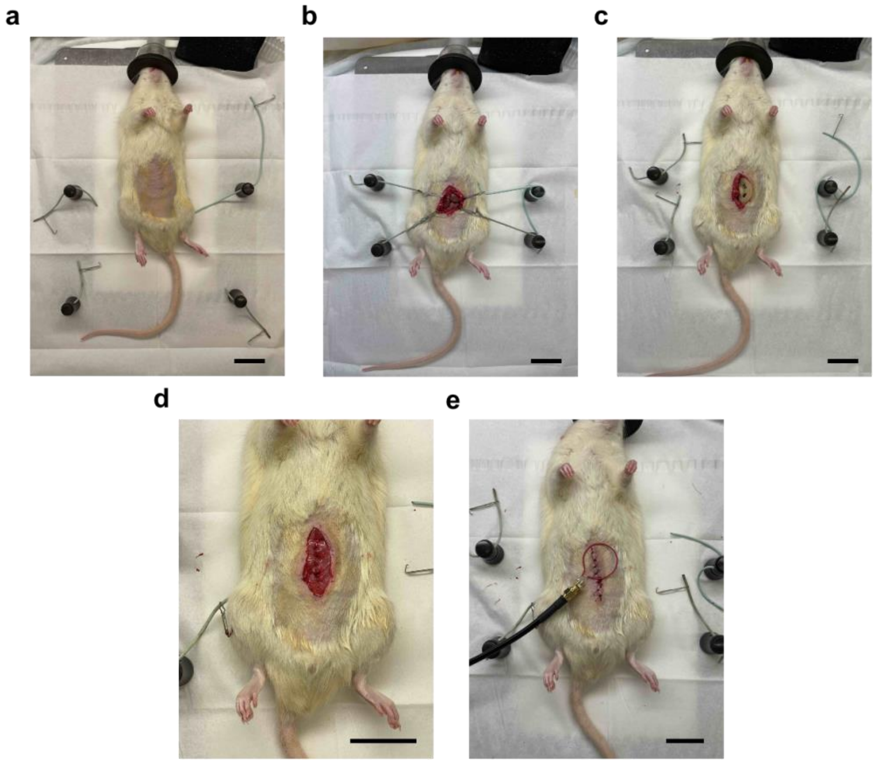

Fig. S28. Surgery and device implantation procedure of the rodent model (rat).

(a) Anesthesia, skin preparation, and sterilization in the supine position. (b) Laparotomy with a 3-cm midline incision on the abdomen. (c) Implantation of the sterilized pH sensor. (d) Abdomen muscle and skin closure. (e) Signal detection after suturing. Scale bars = 3 cm.

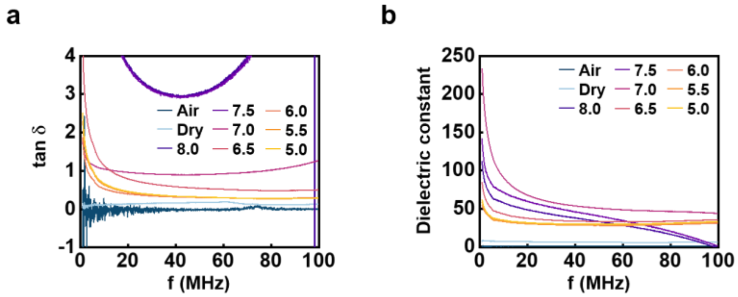

Fig. S29. Dielectric properties of the pH-responsive hydrogel.

(a) Measured loss tangent (tan δ) of air and the pH-responsive hydrogel in the dry state and in swollen states associated with pH buffer solutions. (b) Measured dielectric constant of air and the pH-responsive hydrogel in the dry state and in swollen states associated with pH buffer solutions.

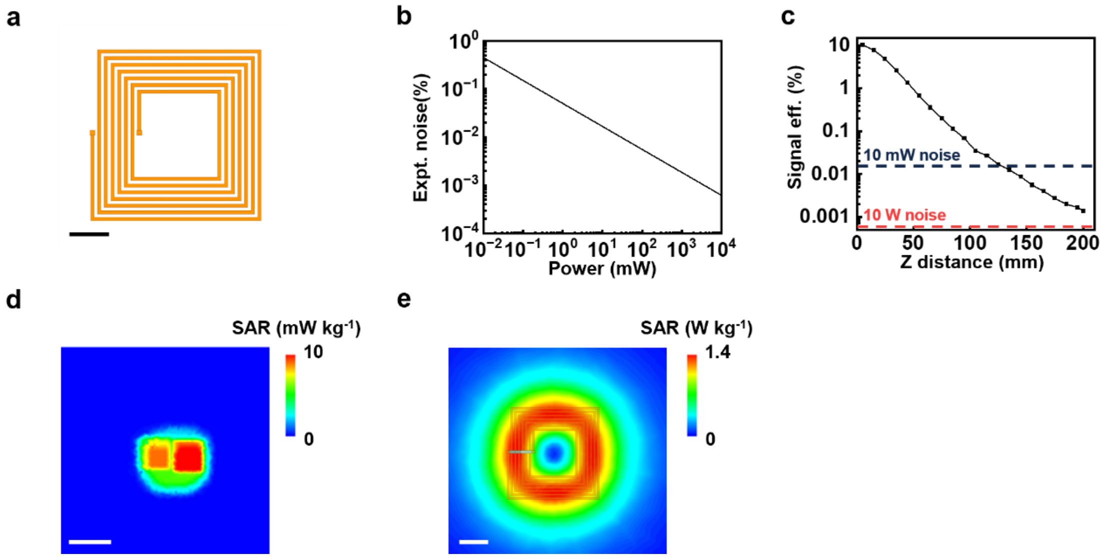

Fig. S30. Characterization of wireless signal detection distance and RF exposure safety.

(a) An improved design of the readout coil for longer signal detection range. (b) Extrapolated experimental noise level as a function of output power of the readout coil. (c) Simulated signal transmission efficiency as a function of separation distance using the improved readout coil. Noise levels with 10 W and 10 mW output power are labeled to compare with signal efficiency at different Z distance. (d) FEA simulated specific absorption rate (SAR) from the device with 1 mW output power and original design of the readout coil. e. FEA simulated SAR from the device with 10 W output power and the improved design of the readout coil. All scale bars = 2 cm.

Table S1. f_s and Q-factors of 3 different pH sensors (as shown in Supplementary Fig. S20).

Sensor #	f_s w/o wax (MHz)	f_s with wax (MHz)	Q-factor w/o wax	Q-factor with wax
1	23.28	23.28	7.54	7.51
2	25.27	25.31	7.00	6.98
3	26.86	26.85	8.50	8.51

REFERENCES

1. T. H. Kim, J. H. Kim, C.-I. Shin, S. H. Kim, J. K. Han, B. I. Choi, CT findings suggesting anastomotic leak and predicting the recovery period following gastric surgery. *Eur. Radiol.* **25**, 1958–1966 (2015).

2. Y.-E. Kim, J. S. Lim, W. J. Hyung, S. K. Lee, J.-Y. Choi, S. H. Noh, M.-J. Kim, K. W. Kim, Clinical implication of positive oral contrast computed tomography for the evaluation of postoperative leakage after gastrectomy for gastric cancer. *J. Comput. Assist. Tomogr.* **34**, 537–542 (2010).

3. R. Gonzalez, M. G. Sarr, D. C. Smith, M. Baghai, M. Kendrick, S. Szomstein, R. Rosenthal, M. M. Murr, Diagnosis and contemporary management of anastomotic leaks after gastric bypass for obesity. *J. Am. Coll. Surg.* **204**, 47–55 (2007).

4. T. Almahmeed, Morbidity of anastomotic leaks in patients undergoing Roux-en-Y gastric bypass. *Arch. Surg.* **142**, 954 (2007).

5. L. J. Greenfield, M. W. Mulholland, *Greenfield's surgery: Scientific principles and practice* editors, Michael W. *Mulholland [and others], with 216 contributors ; illustrations by Holly R. Fischer.* (Lippincott Williams & Wilkins, Philadelphia, PA, 4th ed., 2006).

6. C. Ballesta, R. Berindoague, M. Cabrera, M. Palau, M. Gonzales, Management of anastomotic leaks after laparoscopic Roux-en-Y gastric bypass, *Obes. Surg.* **18**, 623–630 (2008).

7. S. Al-Sabah, M. Ladouceur, N. Christou, Anastomotic leaks after bariatric surgery: It is the host response that matters. *Surg. Obes. Relat. Dis.* **4**, 152–157 (2008).

8. A. Csendes, P. Burdiles, A. M. Burgos, F. Maluenda, J. C. Diaz, Conservative management of anastomotic leaks after 557 open gastric bypasses. *Obes. Surg.* **15**, 1252–1256 (2005).

9. S.-W. Hwang, H. Tao, D.-H. Kim, H. Cheng, J.-K. Song, E. Rill, M. A. Brenckle, B. Panilaitis, S. M. Won, Y.-S. Kim, Y. M. Song, K. J. Yu, A. Ameen, R. Li, Y. Su, M. Yang, D. L.

Kaplan, M. R. Zakin, M. J. Slepian, Y. Huang, F. G. Omenetto, J. A. Rogers, A physically transient form of silicon electronics. *Science* **337**, 1640–1644 (2012).

10. S.-K. Kang, R. K. J. Murphy, S.-W. Hwang, S. M. Lee, D. V. Harburg, N. A. Krueger, J. Shin, P. Gamble, H. Cheng, S. Yu, Z. Liu, J. G. McCall, M. Stephen, H. Ying, J. Kim, G. Park, R. C. Webb, C. H. Lee, S. Chung, D. S. Wie, A. D. Gujar, B. Vemulapalli, A. H. Kim, K.-M. Lee, J. Cheng, Y. Huang, S. H. Lee, P. V. Braun, W. Z. Ray, J. A. Rogers, Bioresorbable silicon electronic sensors for the brain. *Nature* **530**, 71–76 (2016).

11. H. Tao, S.-W. Hwang, B. Marelli, B. An, J. E. Moreau, M. Yang, M. A. Brenckle, S. Kim, D. L. Kaplan, J. A. Rogers, F. G. Omenetto, Silk-based resorbable electronic devices for remotely controlled therapy and in vivo infection abatement. *Proc. Natl. Acad. Sci.* **111**, 17385–17389 (2014).

12. M. Luo, A. W. Martinez, C. Song, F. Herrault, M. G. Allen, A microfabricated wireless RF pressure sensor made completely of biodegradable materials. *J. Microelectromech. Syst.* **23**, 4–13 (2014).

13. W. Bai, J. Shin, R. Fu, I. Kandela, D. Lu, X. Ni, Y. Park, Z. Liu, T. Hang, D. Wu, Y. Liu, C. R. Haney, I. Stepien, Q. Yang, J. Zhao, K. R. Nandoliya, H. Zhang, X. Sheng, L. Yin, K. MacRenaris, A. Brikha, F. Aird, M. Pezhouh, J. Hornick, W. Zhou, J. A. Rogers, Bioresorbable photonic devices for the spectroscopic characterization of physiological status and neural activity. *Nat Biomed Eng.* **3**, 644–654 (2019).

14. L. Yin, H. Cheng, S. Mao, R. Haasch, Y. Liu, X. Xie, S. Hwang, H. Jain, S. Kang, Y. Su, R. Li, Y. Huang, J. A. Rogers, Dissolvable metals for transient electronics. *Adv. Funct. Mater.* **24**, 645–658 (2014).

15. K. J. Yu, D. Kuzum, S.-W. Hwang, B. H. Kim, H. Juul, N. H. Kim, S. M. Won, K. Chiang, M. Trumpis, A. G. Richardson, H. Cheng, H. Fang, M. Thompson, H. Bink, D. Talos, K. J. Seo, H. N. Lee, S.-K. Kang, J.-H. Kim, J. Y. Lee, Y. Huang, F. E. Jensen, M. A. Dichter, T. H. Lucas, J. Viventi, B. Litt, J. A. Rogers, Bioresorbable silicon electronics for transient spatiotemporal mapping of electrical activity from the cerebral cortex. *Nat. Mater.* **15**, 782–791 (2016).

16. J. Shin, Y. Yan, W. Bai, Y. Xue, P. Gamble, L. Tian, I. Kandela, C. R. Haney, W. Spees, Y. Lee, M. Choi, J. Ko, H. Ryu, J.-K. Chang, M. Pezhouh, S.-K. Kang, S. M. Won, K. J. Yu, J. Zhao, Y. K. Lee, M. R. MacEwan, S.-K. Song, Y. Huang, W. Z. Ray, J. A. Rogers, Bioresorbable pressure sensors protected with thermally grown silicon dioxide for the monitoring of chronic diseases and healing processes. *Nat Biomed Eng.* **3**, 37–46 (2019).

17. J. Shin, Z. Liu, W. Bai, Y. Liu, Y. Yan, Y. Xue, I. Kandela, M. Pezhouh, M. R. MacEwan, Y. Huang, W. Z. Ray, W. Zhou, J. A. Rogers, Bioresorbable optical sensor systems for monitoring of intracranial pressure and temperature. *Sci. Adv.* **5**, eaaw1899 (2019).

18. D. Lu, Y. Yan, R. Avila, I. Kandela, I. Stepien, M. Seo, W. Bai, Q. Yang, C. Li, C. R. Haney, E. A. Waters, M. R. MacEwan, Y. Huang, W. Z. Ray, J. A. Rogers, Bioresorbable, wireless, passive sensors as temporary implants for monitoring regional body temperature. *Adv. Healthc. Mater.* **9**, e2000942 (2020).

19. D. Lu, Y. Yan, Y. Deng, Q. Yang, J. Zhao, M. Seo, W. Bai, M. R. MacEwan, Y. Huang, W. Z. Ray, J. A. Rogers, Bioresorbable wireless sensors as temporary implants for in vivo measurements of pressure. *Adv. Funct. Mater.* **30**, 2003754 (2020).

20. Q. Yang, S. Lee, Y. Xue, Y. Yan, T. Liu, S. Kang, Y. J. Lee, S. H. Lee, M. Seo, D. Lu, J. Koo, M. R. MacEwan, R. T. Yin, W. Z. Ray, Y. Huang, J. A. Rogers, Materials, mechanics designs, and bioresorbable multisensor platforms for pressure monitoring in the intracranial space. *Adv. Funct. Mater.* **30**, 1910718 (2020).

21. C. M. Boutry, L. Beker, Y. Kaizawa, C. Vassos, H. Tran, A. C. Hinckley, R. Pfattner, S. Niu, J. Li, J. Claverie, Z. Wang, J. Chang, P. M. Fox, Z. Bao, Biodegradable and flexible arterial-pulse sensor for the wireless monitoring of blood flow. *Nat. Biomed. Eng.* **3**, 47–57 (2019).

22. A. Palmroth, T. Salpavaara, P. Vuoristo, S. Karjalainen, T. Kääriäinen, S. Miettinen, J. Massera, J. Lekkala, M. Kellomäki, Materials and orthopedic applications for bioresorbable inductively coupled resonance sensors. *ACS Appl. Mater. Interfaces* **12**, 31148–31161 (2020).

23. S.-W. Hwang, C. H. Lee, H. Cheng, J.-W. Jeong, S.-K. Kang, J.-H. Kim, J. Shin, J. Yang, Z. Liu, G. A. Ameer, Y. Huang, J. A. Rogers, Biodegradable elastomers and silicon nanomembranes/nanoribbons for stretchable, transient electronics, and biosensors. *Nano Lett.* **15**, 2801–2808 (2015).

24. M. Corsi, A. Paghi, S. Mariani, G. Golinelli, A. Debrassi, G. Egri, G. Leo, E. Vandini, A. Vilella, L. Dähne, D. Giuliani, G. Barillaro, Bioresorbable nanostructured chemical sensor for monitoring of pH level in vivo. *Adv. Sci.* **9**, e2202062 (2022).

25. X. Zhao, X. Chen, H. Yuk, S. Lin, X. Liu, G. Parada, Soft materials by design: Unconventional polymer networks give extreme properties. *Chem. Rev.* **121**, 4309–4372 (2021).

26. X. Liu, J. Liu, S. Lin, X. Zhao, Hydrogel machines. *Mater. Today* **36**, 102–124 (2020).

27. C. Yang, Z. Suo, Hydrogel ionotronics, *Nat Rev Mater*. **3**, 125–142 (2018).

28. K. Unger, F. Greco, A. M. Coclite, Temporary tattoo pH sensor with pH-responsive hydrogel via initiated chemical vapor deposition. *Adv. Mater. Technol.* **7**, 2100717 (2022).

29. L. Klouda, A. G. Mikos, Thermoresponsive hydrogels in biomedical applications. *Eur. J. Pharm. Biopharm.* **68**, 34–45 (2008).

30. L. Li, J. M. Scheiger, P. A. Levkin, Design and applications of photoresponsive hydrogels. *Adv. Mater.* **31**, e1807333 (2019).

31. P. Rahmani, A. Shojaei, A review on the features, performance and potential applications of hydrogel-based wearable strain/pressure sensors. *Adv. Colloid Interface Sci.* **298**, 102553 (2021).

32. N. A. Peppas, J. Z. Hilt, A. Khademhosseini, R. Langer, Hydrogels in biology and medicine: From molecular principles to bionanotechnology. *Adv. Mater.* **18**, 1345–1360 (2006).

33. M. C. Koetting, J. T. Peters, S. D. Steichen, N. A. Peppas, Stimulus-responsive hydrogels: Theory, modern advances, and applications. *Mater. Sci. Eng.: R: Rep.* **93**, 1–49 (2015).

34. F. Pinelli, L. Magagnin, F. Rossi, Progress in hydrogels for sensing applications: A review. *Mater Today Chem.* **17**, 100317 (2020).

35. H. M. El-Husseiny, E. A. Mady, L. Hamabe, A. Abugomaa, K. Shimada, T. Yoshida, T. Tanaka, A. Yokoi, M. Elbadawy, R. Tanaka, Smart/stimuli-responsive hydrogels: Cutting-edge platforms for tissue engineering and other biomedical applications. *Mater Today Bio.* **13**, 100186 (2022).

36. J. Himpens, J. Dobbeleir, G. Peeters, Long-term results of laparoscopic sleeve gastrectomy for obesity. *Ann. Surg.* **252**, 319–324 (2010).

37. D. Nguyen, F. Dip, L. Hendricks, E. Lo Menzo, S. Szomstein, R. Rosenthal, The surgical management of complex fistulas after sleeve gastrectomy. *Obes. Surg.* **26**, 245–250 (2016).

38. N. Deirram, C. Zhang, S. S. Kermaniyan, A. P. R. Johnston, G. K. Such, pH-responsive polymer nanoparticles for drug delivery. *Macromol. Rapid Commun.* **40**, 1800917 (2019).

39. S. Chaterji, I. K. Kwon, K. Park, Smart polymeric gels: Redefining the limits of biomedical devices. *Prog. Polym. Sci.* **32**, 1083–1122 (2007).

40. H. Fan, P. Li, W. Li, H. Li, X. Huang, Ultrasensitive (co) polymers based on poly (methacrylamide) structure with fining-tunable pH responsive value. *Molecules* **23**, 1870 (2018).

41. M. B. Browning, S. N. Cereceres, P. T. Luong, E. M. Cosgriff-Hernandez, Determination of the in vivo degradation mechanism of PEGDA hydrogels. *J. Biomed. Mater. Res. A* **102A**, 4244–4251 (2014).

42. B. Reid, M. Gibson, A. Singh, J. Taube, C. Furlong, M. Murcia, J. Elisseeff, PEG hydrogel degradation and the role of the surrounding tissue environment. *J Tissue Eng. and Regener. Med.* **9**, 315–318 (2013).

43. A. D. Lynn, T. R. Kyriakides, S. J. Bryant, Characterization of the in vitro macrophage response and in vivo host response to poly(ethylene glycol)-based hydrogels. *J. Biomed. Mater. Res., Part A* **3**, 941–953 (2009).

44. R. C. Webb, A. P. Bonifas, A. Behnaz, Y. Zhang, K. J. Yu, H. Cheng, M. Shi, Z. Bian, Z. Liu, Y.-S. Kim, W.-H. Yeo, J. S. Park, J. Song, Y. Li, Y. Huang, A. M. Gorbach, J. A. Rogers, Ultrathin conformal devices for precise and continuous thermal characterization of human skin. *Nat. Mater.* **12**, 938–944 (2013).

45. S. M. Won, J. Koo, K. E. Crawford, A. D. Mickle, Y. Xue, S. Min, L. A. McIlvried, Y. Yan, S. B. Kim, S. M. Lee, B. H. Kim, H. Jang, M. R. MacEwan, Y. Huang, R. W. Gereau, J. A. Rogers, Natural wax for transient electronics. *Adv. Funct. Mater.* **28**, 1801819 (2018).

46. Q.-A. Huang, L. Dong, L.-F. Wang, LC passive wireless sensors toward a wireless sensing platform: Status, prospects, and challenges. *J. Microelectromech. Syst.* **25**, 822–841 (2016).

47. C.-C. Peng, A. Chauhan, Ion transport in silicone hydrogel contact lenses. *J Memb Sci.* **399–400**, 95–105 (2012).

48. S. K. De, N. R. Aluru, B. Johnson, W. C. Crone, D. J. Beebe, J. Moore, Equilibrium swelling and kinetics of pH-responsive hydrogels: Models, experiments, and simulations. *J. Microelectromech. Syst* **11**, 544–555 (2002).

49. P. Riddle, pH meters and their electrodes: Calibration, maintenance and use. *Biomed. Sci.*, **202–205** (2013).

50. Q. Shi, H. Liu, D. Tang, Y. Li, X. Li, F. Xu, Bioactuators based on stimulus-responsive hydrogels and their emerging biomedical applications. *NPG Asia Mater.* **11**, 64 (2019).

51. L. Zhu, S. Powell, S. G. Boyes, Synthesis of tertiary amine-based pH-responsive polymers byRAFT Polymerization. *J. Polym. Sci. A Polym. Chem.* **53**, 1010–1022 (2015).

52. H. K. Makadia, S. J. Siegel, Poly lactic-co-glycolic acid (PLGA) as biodegradable controlled drug delivery carrier. *Polymers (Basel).* **3**, 1377–1397 (2011).

53. E. Chiellini, A. Corti, S. D'Antone, R. Solaro, Biodegradation of poly (vinyl alcohol) based materials. *Prog. Polym. Sci.* **28**, 963–1014 (2003).

54. Y. Zhang, H. Fu, Y. Su, S. Xu, H. Cheng, J. A. Fan, K.-C. Hwang, J. A. Rogers, Y. Huang, Mechanics of ultra-stretchable self-similar serpentine interconnects. *Acta Mater.* **61**, 7816–7827 (2013).

55. S. S. Mohan, M. del Mar Hershenson, S. P. Boyd, T. H. Lee, Simple accurate expressions for planar spiral inductances. *IEEE J Solid-State Circuits.* **34**, 1419–1424 (1999).

56. D. Lu, S. Li, Q. Yang, H. M. Arafa, Y. Xu, Y. Yan, D. Ostojich, W. Bai, H. Guo, C. Wu, S. Li, L. Jacobson, A. M. Westman, M. R. MacEwan, Y. Huang, M. Pet, J. A. Rogers, Implantable, wireless, self-fixing thermal sensors for continuous measurements of microvascular blood flow in flaps and organ grafts. *Biosens. Bioelectron.* **206**, 114145 (2022).

57. J. Kim, D. Azagury, D. Eisenberg, E. DeMaria, G. M. Campos, ASMBS position statement on prevention, detection, and treatment of gastrointestinal leak after gastric bypass and sleeve gastrectomy, including the roles of imaging, surgical exploration, and nonoperative management. *Surg. Obes. Relat. Dis.* **11**, 739–748 (2015).

58. A. A. Rached, Gastric leaks post sleeve gastrectomy: Review of its prevention and management. *World J. Gastroenterol.* **20**, 13904 (2014), 13910.

59. L. L. Zheng, V. Vanchinathan, R. Dalal, J. Noolandi, D. J. Waters, L. Hartmann, J. R. Cochran, C. W. Frank, C. Q. Yu, C. N. Ta, Biocompatibility of poly(ethylene glycol) and poly (acrylic acid) interpenetrating network hydrogel by intrastromal implantation in rabbit cornea, *J. Biomed. Mater. Res. A* **103**, 3157–3165 (2015).

60. M. Dautta, M. Alshetaiwi, A. Escobar, F. Torres, N. Bernardo, P. Tseng, Multi-functional hydrogel-interlayer RF/NFC resonators as a versatile platform for passive and wireless biosensing. *Adv Electron Mater.* **6**, 1901311 (2020).

61. M. Dautta, M. Alshetaiwi, J. Escobar, P. Tseng, Passive and wireless, implantable glucose sensing with phenylboronic acid hydrogel-interlayer RF resonators. *Biosens. Bioelectron.* **151**, 112004 (2020).

62. A. A. La Mattina, S. Mariani, G. Barillaro, Bioresorbable materials on the rise: From electronic components and physical sensors to in vivo monitoring systems. *Adv. Sci.* **7**, 1902872 (2020).

63. N. E. Aranda-Ledesma, I. Bautista-Hernández, R. Rojas, P. Aguilar-Zárate, N. D. P. Medina-Herrera, C. Castro-López, G. C. Guadalupe Martínez-Ávila, Candelilla wax: Prospective suitable applications within the food field. *LWT.* **159**, 113170 (2022).

64. A. Mandal, J. R. Clegg, A. C. Anselmo, S. Mitragotri, Hydrogels in the clinic. *Bioeng. Transl. Med.* **5**, e10158 (2020).

65. J. R. Choi, K. W. Yong, J. Y. Choi, A. C. Cowie, Recent advances in photo-crosslinkable hydrogels for biomedical applications. *Biotechniques* **66**, 40–53 (2019).

66. A. S. M. Wong, S. K. Mann, E. Czuba, A. Sahut, H. Liu, T. C. Suekama, T. Bickerton, A. P. R. Johnston, G. K. Such, Self-assembling dual component nanoparticles with endosomal escape capability. *Soft Matter* **11**, 2993–3002 (2015).

67. V. Kalidasan, X. Yang, Z. Xiong, R. R. Li, H. Yao, H. Godaba, S. Obuobi, P. Singh, X. Guan, X. Tian, S. A. Kurt, Z. Li, D. Mukherjee, R. Rajarethinam, C. S. Chong, J.-W. Wang, P. L. R. Ee, W. Loke, B. C. K. Tee, J. Ouyang, C. J. Charles, J. S. Ho, Wirelessly operated bioelectronic sutures for the monitoring of deep surgical wounds. *Nat. Biomed. Eng.* **5**, 1217–1227 (2021).

68. J. Li, D. J. Mooney, Designing hydrogels for controlled drug delivery. *Nat. Rev. Mater.* **1**, 16071 (2016).

69. J. Liu, T.-M. Fu, Z. Cheng, G. Hong, T. Zhou, L. Jin, M. Duvvuri, Z. Jiang, P. Kruskal, C. Xie, Z. Suo, Y. Fang, C. M. Lieber, Syringe-injectable electronics. *Nat. Nanotechnol.* **10**, 629–636 (2015).

70. X. Chen, C. Dong, K. Wei, Y. Yao, Q. Feng, K. Zhang, F. Han, A. F.-T. Mak, B. Li, L. Bian, Supramolecular hydrogels cross-linked by preassembled host–guest PEG cross-linkers resist excessive, ultrafast, and non-resting cyclic compression, *NPG Asia Mater.* **10**, 788–799 (2018).

71. M. Stanculescu, L. Iordache, M. Iordache, D. Niculae, V. Bucata, Using S parameters in wireless power transfer analysis. *10th International Symposium on Advanced Topics in Electrical Engineering*, 107–112 (2017).

72. Wireless Devices and Health Concerns. https://fcc.gov/consumers/guides/wireless-devices-and-health-concerns (2020).
